# Three new species and one new subspecies of Depressariinae (Lepidoptera) from Europe

**DOI:** 10.3897/zookeys.684.13383

**Published:** 2017-07-12

**Authors:** Peter Buchner, Martin Corley, Jari Junnilainen

**Affiliations:** 1 Scheibenstraße 335, 2625 Schwarzau am Steinfeld, Austria; 2 Pucketty Farm Cottage, Faringdon, Oxfordshire SN7 8JP, United Kingdom; 3 CIBIO, Centro de Investigação em Biodiversidade e Recursos Genéticos, Universidade do Porto, Campus Agrário de Vairão, P-4485–661 Vairão, Portugal; 4 Mahlapolku 3, 01730 Vantaa, Finland

**Keywords:** Lepidoptera, Gelechioidea, Depressariidae, *Depressaria*, *Agonopterix*, Italy, Greece, Morocco, Portugal, Spain, Canary Islands, new species, DNA barcoding

## Abstract

The species *Depressaria albarracinella* Corley, **sp. n.**, *Agonopterix carduncelli* Corley, **sp. n.** and *Agonopterix pseudoferulae* Buchner & Junnilainen, **sp. n.** and the subspecies Depressaria saharae Gastón & Vives ssp. tabelli Buchner, **ssp. n.** are described.

*Depressaria albarracinella* was first found in Spain in 1969 and recognised as apparently new but the specimens in NHMUK have remained undescribed. Additional Spanish material has been located in ZMUC and other collections and three specimens have been found from Greece.

*Agonopterix carduncelli*. A single male of an unidentified *Agonopterix* of the *pallorella* group was found in Algarve, Portugal in 2010. A search for larvae in March 2011 was successful and one male and one female were reared from *Carthamus caeruleus*. Additional specimens of the new species have been located in collections from Spain, Greece and Morocco.

*Agonopterix pseudoferulae*. A specimen from Greece with the name *Agonopterix ferulae* (Zeller, 1847) found in the Klimesch collection in ZSM had forewing markings which suggested that it might be a different species. Further specimens from Italy and Greece have been examined, among them two reared from *Elaeoselinum asclepium* (Apiaceae). Both genitalia and barcode show that this is an undescribed species.

*Depressaria saharae* Gastón & Vives, 2017 was described very recently (Gastón and Vives 2017) from northern Spain with a brief description, and figures of two males and male genitalia. Here the new species is redescribed, and additional data on distribution and relationships of the new species added. The opportunity is also taken to show that Canary Islands specimens with the same male genitalia should be treated as a new subspecies D. saharae ssp. tabelli Buchner, **ssp. n.**

## Introduction

Preparatory work for a proposed volume on Depressariinae in the series *Microlepidoptera of Europe* has revealed a number of taxonomically challenging species groups in the genera *Agonopterix* Hübner [1825] and *Depressaria* Haworth, 1811. This was not unexpected but there are more such groups than we had initially expected. However, in addition to the problem groups, some undescribed species have also been discovered which can be described without the necessity to resolve complex taxonomic issues. Two such species are described here in *Agonopterix* and one species and one subspecies in *Depressaria*.

## Material and methods

**Material** has been examined from NHMUK, NHMV, TLMF, ZMUC, ZSM and additionally specimens from many private collectors including those of the authors have been checked, here only listed if the material was of particular importance for this paper: Michael Dale (England), Gabriele Fiumi (Italy), Knud Larsen (Denmark), Toni Mayr (Austria), Willibald Schmitz (Germany), Peter Sonderegger (Switzerland), Lubomír Srnka (Slovakia), Jan Šumpich (Czech Republic) and Joachim Viehmann (Germany). Apart from one exception given in the description, each species includes both reared and light-trapped specimens.

**Morphological examination and photographic documentation.** Genitalia preparations followed standard techniques (Robinson 1976). Male preparations were stained with mercurochrome and females with chlorazol. The placement of holotypes is given under each species. Photographic documentation: Apart from two exceptions given in the descriptions, photos of specimens in total view were taken with Canon EOS 5D Mark III and Canon lens EF 100mm 2.8 L IS USM at 1:1., specimens were illuminated with two diffused flashes, using a third flash for setting the background whiteness. Detailed photos of specimens were taken with a Canon lens MP-E 65 at 2:1, using ring flash. Genitalia photos were taken with microscope (Wild Heerbrugg) using a 10x objective and a 2.5x ocular. Photos were edited using the software Helicon Focus 4.80 and Adobe Photoshop 6.0. For creating the black and white photos, based on the used stain, the G alpha channel of the RGB originals was used in males and the Y alpha channel of the CMYK originals in females. Genitalia examination and photos by P. Buchner, if not specified.

**DNA-Barcoding.** The full length lepidopteran DNA barcode sequence is a 658 basepair long segment of the 5’ terminus of the mitochondrial COI gene (cytochrome c oxidase 1). DNA samples (dried leg) were prepared according to the accepted standards and were processed at the Canadian Centre for DNA Barcoding (CCDB, Biodiversity Institute of Ontario, University of Guelph) to obtain DNA barcodes using the standard high-throughput protocol described in deWaard et al. (2008). Detailed specimen data are listed under Genetic data of species description. Sequences were submitted to GenBank: accession numbers are listed in an Appendix. Further details including complete voucher data and images can be accessed in the public dataset DS-DEEUR330 (http://www.boldsystems.org/index.php/Public_SearchTerms?query=DS-DEEUR330, dx.doi.org/10.5883/DS-DEEUR330) in the Barcode of Life Data Systems (BOLD; Ratnasingham and Hebert 2007). Neighbour-joining trees of DNA barcode data were constructed using Mega 5 (Tamura et al. 2011) under the Kimura 2 parameter model for nucleotide substitutions. Additional, the evolutionary history was inferred by using the Maximum Likelihood method based on the Tamura-Nei model. Evolutionary analyses were conducted in MEGA7. This result is shown in radiation graphic, because in this view the evolutionary aspect is visualized better than in traditional tree.

### Abbreviations

**DEEUR** “Depressariinae of Europe”, prefix for number of a photo or slide made by P. Buchner

**MNHN** Muséum National d’Histoire Naturelle, Paris, France

**NHMUK** (formerly **BMNH)** Natural History Museum (British Museum, Natural History), London, United Kingdom

**NHMV** Naturhistorisches Museum, Vienna, Austria

**NMPC** National Museum, Prague, Czech Republic

**TLMF** Tiroler Landesmuseum Ferdinandeum, Innsbruck, Austria

**ZMUC** Zoological Museum, University of Copenhagen, Denmark

**ZMUH** Zoology Museum, University of Helsinki, Finland

**ZSM** Zoologische Staatssammlung München, Germany

## Description of new species

### Depressaria albarracinella

Taxon classificationAnimaliaLepidopteraDepressariidae

Corley sp. n.

http://zoobank.org/69805CE8-47FC-43FA-BE58-06F6DB16C946

#### Type locality.

Spain, Granada, Sierra Nevada, Collado del Lobo, north side, 2300 m.

#### Holotype.

♂ Sierra Nevada, Collado del Lobo, North Side, 2300 m, 14.vii.1969 | Hispania mer. K. Sattler & D.J. Carter. BM 1970-26 | HOLOTYPE *Depressaria albarracinella* Corley, teste M. Corley, 2004 | B.M. ♂ Genitalia slide No. 30716 | Corley prep. 1915m.

#### Paratypes.

**Spain**: ♀ Sierra Nevada, Collado del Lobo, North Side, 2300 m, 14.vii.1969, Hisp. mer. K. Sattler & D.J. Carter. BM 1970-26, *Depressaria albarracinella* Corley, det. M. Corley, 2004; ♂ Prov. Granada, Sierra Nevada, Puerto de la Ragua, 1000m, 1.vii.1969 K. Sattler & D.J. Carter. NHMUK prep. 18856 (NHMUK); ♀ Andalusia, Sierra Nevada, Camina de Veleta, 2300 m, 23.x.1983, leg. E. Traugott-Olsen (ZMUC); 6 ♂♂, 2 ♀♀, Spain, Almería, Sierra de los Filabres, Alto del Calar del Gallinero, 1900–2022m, 17.-18.vi.2007, J. Šumpich leg. et det. (NMPC); 2 ♀♀, Spain, Almería, Sierra de los Filabres, route Purchena – Senés, 1600m, 16.vi.2007, J. Šumpich leg. et det. (NMPC); ♂ Castellón, Banderetta Pass, 800 m, 17.vii.1992, leg. M. Fibiger (ZMUC); ♂ Teruel, Albarracin, Val de Vecar, 1250 m, 17–18.vii.1981, leg. M. Fibiger (ZMUC) (Corley gen. prep. 1711); ♀ Teruel, Albarracin, Val de Vecar, 15.vii.1992. M. Fibiger (ZMUC) (Corley gen. prep. 1717); ♂ Teruel, Albarracin, 1150 m, 3.v.2002, leg. K. Černý, det. P. Buchner; ♀ Zaragosa, Bujareloz, 6 km, ♀, 300m 29.v.2015, leg. J. Viehmann, det P. Buchner; ♂ Huesca, Candasnos, 10 km S, 30.v.2015, leg. J. Viehmann, det P. Buchner.

#### Other material examined.

**Greece**: ♂ Central Greece, Parnassos Mountains, 1 km NE Arachova, 1070 m, 9.vi.2013, leg. P. Skou (ZMUC), det. P. Buchner; 2♂♂ Lesbos, Molivos, 6.vi.1994 (gen.prep. DEEUR 5398) and 7.vi.1994, leg. J.P. Baungaard (ZMUC), det. P. Buchner

#### Diagnosis.

Externally *D. albarracinella* differs from other species of the *veneficella* group in the very weak or obsolete dark forewing markings and the absence of a dark spot at base of dorsum, but it is more reliably separated from other species in the group by various characters involving different proportions of one part of the male genitalia relative to another. This is best set out in a key.

The key below includes only the European species. *D. pentheri* Rebel, 1904 is omitted due to insufficient knowledge of this taxon. The North African *D. deverrella* Chrétien, 1915, has sometimes been listed as present in France, but we can find no evidence for this.

**Figure 1.**
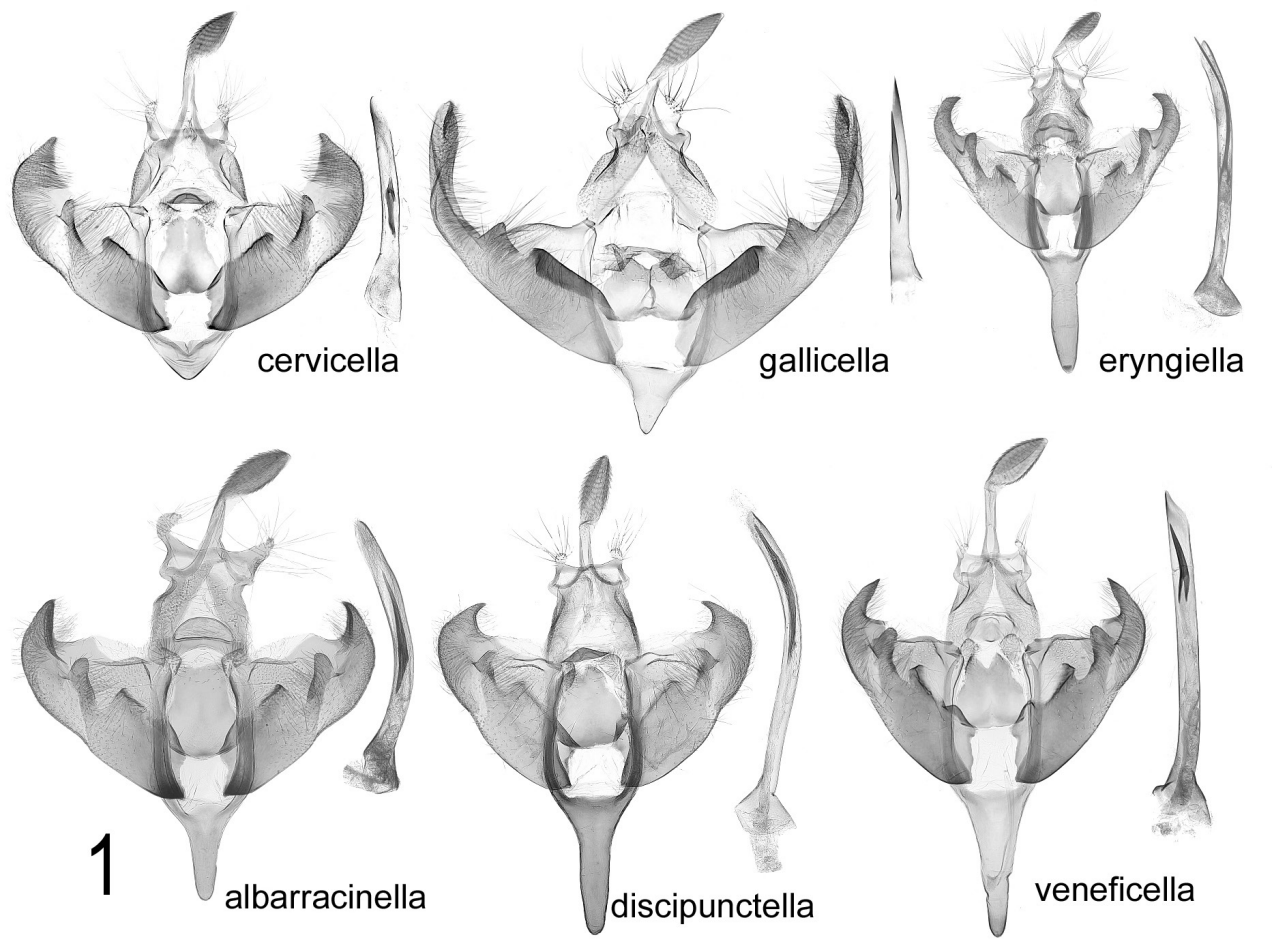
Comparison of male genitalia of European *D. veneficella* group species. *D. cervicella* (Austria, Mödling) *D. gallicella* (Switzerland, Saillon) *D. eryngiella* (Turkey, Malaty, Murhak Dagh) *D. albarracinella* sp. n. (Greece, Arachova) *D. discipunctella* (Macedonia, Petrina) *D. veneficella* (Italy, Sicily).

**Key to males of European species of *Depressaria veneficella* group (see comparison in Fig. 1)**

**Table:** 

1	Saccus very short, not exceeding one quarter of valva length	**2**
–	Saccus clearly longer than one quarter of valva	**3**
2	Aedeagus shorter than valva; valva nearly parallel-sided in distal two-fifths, slender, blunt	***gallicella* Chrétien, 1908**
–	Aedeagus about as long as valva, valva tapering to a sharp tip	***cervicella* Herrich-Schäffer, 1854**
3	Cornutus short, shorter than one-third of aedeagus	***veneficella* Zeller, 1847**
–	Cornutus longer than one-third of aedeagus	**4**
4	Saccus less than half as long as valva	***albarracinella* Corley, sp. n.**
–	Saccus longer, more than half as long as valva	**5**
5	Distal part of valva, beyond median bulge, slender, length to width ratio of this part 3:1 or more	***eryngiella* Millière, 1881**
–	Distal part of valva, beyond median bulge, rapidly tapering from wide base, length to width ratio of this part 2:1 or less	***discipunctella* Herrich-Schäffer, 1854**

**Key to females of European species of *Depressaria veneficella* group**

**Table:** 

1	Ductus bursae expanded at anterior end then twisted and finally constricted at entrance to corpus bursae	**2**
–	Ductus bursae simple without constriction at entrance to corpus bursae	**4**
2	Ostium close to posterior margin of sternite VIII	**3**
–	Ostium opening on margin of sternite VIII	***eryngiella* Millière, 1881**
3	Sternite VIII anteriorly with a pair of sclerotized cusps, on either side of antrum	***veneficella* Zeller, 1847**
–	Sternite VIII without such cusps	***discipunctella* Herrich-Schäffer, 1854**
4	Anterior margin of sternite VIII with deep sinus; ostium close to posterior margin	***albarracinella* Corley, sp. n.**
–	Anterior margin of sternite VIII straight or slightly convex	**5**
5	Signum minute; ductus bursae of uniform width	***gallicella* Chrétien, 1908**
–	Signum small, but wider than narrowest part of ductus bursae close to antrum; ductus bursae with swelling in middle	***cervicella* Herrich-Schäffer, 1854**

**Figure 2.**
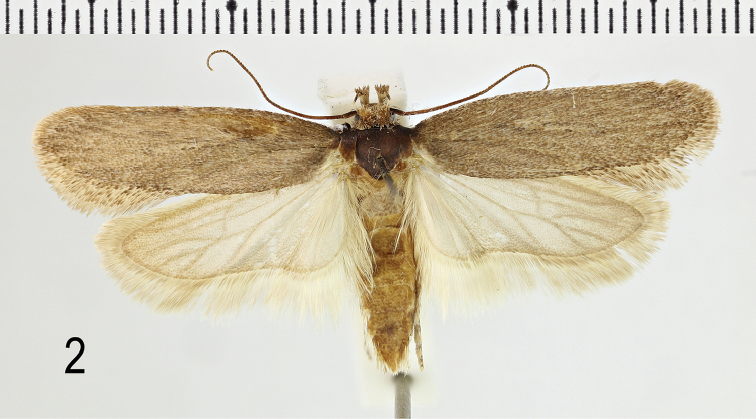
*Depressaria albarracinella* sp. n. Holotype male, Spain, Granada, Sierra Nevada, Collado del Lobo, North Side, 2300 m, 14.vii.1969, leg. K. Sattler & D.J. Carter (NHMUK) (photo D. Lees).

**Figures 3–4.**
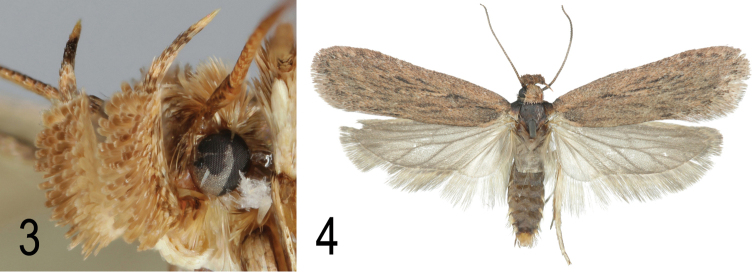
**3** *Depressaria albarracinella* sp. n. Head. Paratype. Spain, Granada, Sierra Nevada, Camina de Veleta, 2300 m, 22.x.1987, leg. E. Traugott-Olsen (ZMUC) **4** *Depressaria albarracinella* sp. n. Paratype. Spain, Huesca, Candasnos, 30.v.2015, leg. J. Viehmann.

**Description. Adult** (Figs 2–4). Wingspan 23–26 mm. Head cinnamon-brown on neck and crown; face light brownish buff. Labial palp with segment 3 two-thirds length of segment 2, segment 2 buff with tufted scales on ventral side cinnamon; segment 3 cinnamon with dark grey ring beyond middle, tip cinnamon-buff. Antenna light grey-brown, narrowly ringed dark brown. Thorax light brownish buff, rarely darker. Forewing light brown, often with slight cinnamon tinge, often very weakly marked but sometimes with more or less faint grey-brown interrupted streaks in cell, in fold, beyond cell, between veins to costa and between veins to termen; occasionally a faint brown spot is present at base of dorsum; equally indistinct grey-brown spots between vein-ends at termen; cilia light brown, without obvious cilia line. Hindwing light grey, slightly darker posteriorly, with narrow grey-brown line around terminal and dorsal margins; cilia light grey-brown at apex to almost white at dorsal base, with a fine darker cilia line. Abdomen light grey-brown.

Variation: The forewing markings vary from almost completely obsolete to present but faint compared with most other *Depressaria* species. The specimen from Huesca, Spain (Fig. 4) is the most strongly marked that we have seen. Sometimes a faint V-shaped pale fascia is visible beyond end of cell.

**Figures 5–6.**
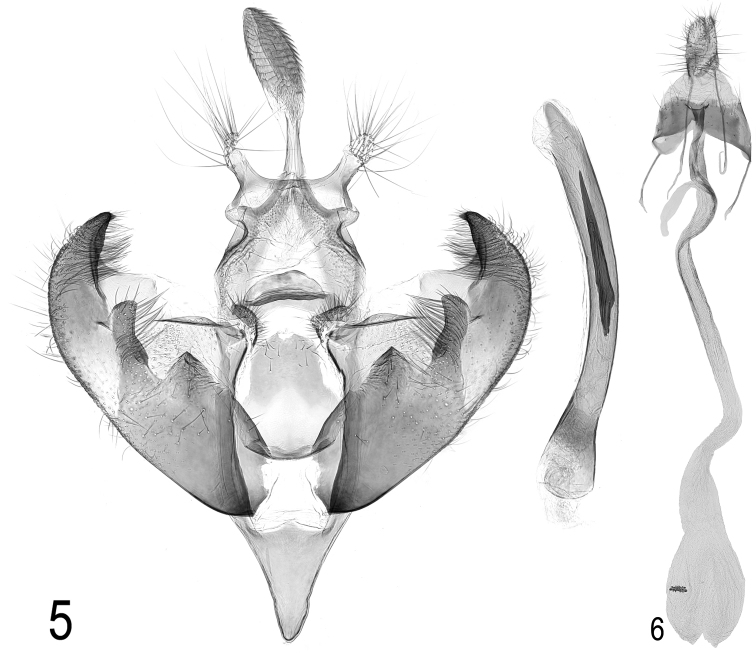
**5** *Depressaria albarracinella* sp. n. Male genitalia. Paratype. Spain, Castellón, Banderetta Pass, 800 m, 17.vii.1992, leg. M. Fibiger, slide DEEUR 0762 (ZMUC) **6** *Depressaria albarracinella* sp. n. Female genitalia. Paratype. Spain, Granada, Camina de Veleta, 2300m, 22.x.1987, leg. E. Traugott-Olsen, slide DEEUR 0772 (ZMUC).

**Male genitalia** (Fig. 5). Gnathos elongate; socii elongate, parallel-sided, divergent; valva almost as long as aedeagus, apex incurved, sacculus with two lobes, the inner broadly triangular, the second longer and narrower, slightly incurved; anellus broadly pyriform, distal margin slightly emarginated; saccus triangular, of similar length to anellus; aedeagus slender with slightly expanded base, cornutus about two-fifths length of aedeagus.

**Female genitalia** (Fig. 6). Anterior margin of sternite VIII with median sinus, ostium close to posterior margin; ductus bursae long, without swellings or ornamentation; signum small, wider than long, not as wide as ductus bursae in most of its length.

#### Molecular data.

**Data of barcoded specimens.** TLMF Lep 19062 (658 bp.[1n], ♀, Spain, Aragon, Albarracin, 40°25'N; 1°27'W, 3.v.2003, leg. et coll. K. Cerny, gen. prep. DEEUR 1786); TLMF Lep 19150 (658 bp.[0n], ♂, Spain, Huesca, Candasnos, 41°30'N; 0°40'E, 30.v.2015, leg. J. Viehmann, coll. W. Schmitz, gen. prep. DEEUR 3903); TLMF Lep 17687 (658 bp.[0n], ♂, Greece, Parnassos Mountains, 1 km NW Arachova, 1070 m, 38°29'N; 22°35'E, 9.vi.2013, leg. P. Skou, coll. ZMUC, gen. prep. DEEUR 2326).

**Figure 7.**
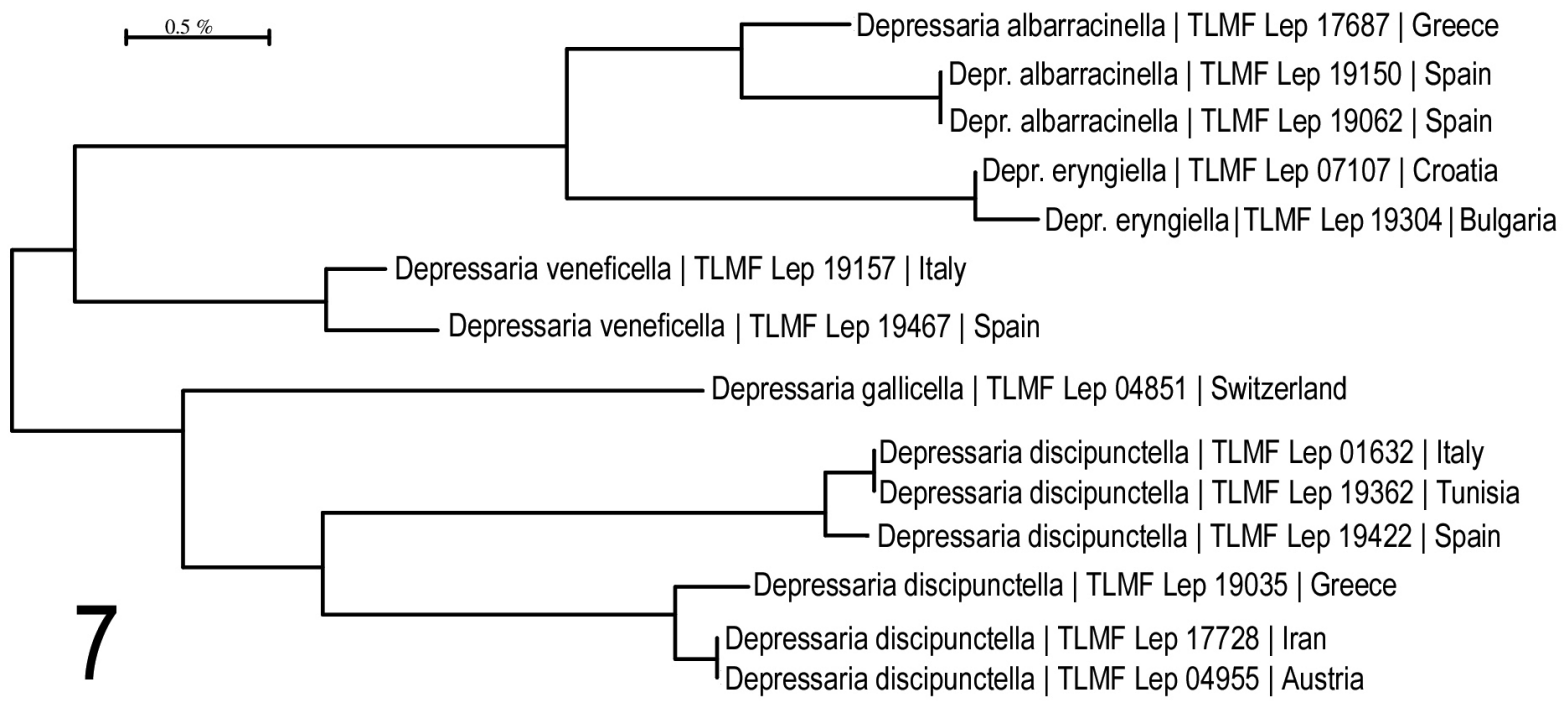
Neighbour-joining tree of *Depressaria albarracinella* sp. n. and related species. Associated BOLD BINs: *D. albaracinella*: BOLD:ACX8130; *D. eryngiella*: BOLD:ACF7124; *D. veneficella*: BOLD:ADC7254; *D. gallicella*: BOLD:ABA1484; *D. discipunctella*: BOLD:AAO4681 (upper cluster) & BOLD:ABA1412 (lower cluster).

**Neighbour-joining analysis** (Fig. 7) shows *Depressaria eryngiella* as the nearest neighbour with 2.45% p-distance. Intraspecific variability, based on present knowledge, 0% within the Spanish population and 1.08% between Spanish and Greek populations.

For Maximum Likelihood analysis, see Fig. 70.

#### Etymology.

The species name is an adjective derived from Albarracin in Spain, an area where two of the paratypes were taken.

#### Distribution.

**Spain**: Mountain areas of Eastern Spain from Sierra Nevada and Sierra de Los Filabres northwards, in the provinces of Granada, Almería, Castellón, Teruel, Zaragosa and Huesca. **Greece**: Parnassos Mountains in Central Greece, Lesbos.

#### Bionomics.

Larva and food-plant unknown, but the latter is likely to belong to Apiaceae. Adult moths have been taken in May, June, July and October. It is probable that overwintering takes place in the adult stage, but less clear when the larvae would be feeding.

#### Remarks.

The genus *Depressaria* Haworth, 1811 includes around 125 species (Wikipedia 2016) with the greatest number in the Palaearctic region. The majority of the species are rather similar externally, but for the most part, the male genitalia give clear differences between species. Indeed there is such diversity in genital morphology within the genus that it is difficult to characterise the genus using genitalia characters. Within this great diversity there are some clearly defined groups, with a number of species sharing a suite of genitalia characters. One such group is the *veneficella* group (Hannemann, 1953), currently with 13 species described from the Palaearctic region extending from western Europe and North Africa through the Middle East to central Asia, with the most eastern records from north-east China, Mongolia and the Altai region of Siberia. Lvovsky (1996) when describing *D. erzurumella* Lvovsky, 1996 from Turkey, provided a key based on male genitalia to 11 species, omitting *D. pentheri* Rebel, 1904, which was known only from the female. Subsequently he described *D. kailai* Lvovsky, 2009 (Lvovsky 2009). The new species, *D. albarracinella*, belongs to this group.

The *veneficella* group is characterised by rather long wings, forewings brown with pattern usually consisting of blackish streaks between the veins, but the pattern very reduced in some species. Male genitalia have elongate gnathos (nearly globose only in *altaica* Zeller, 1854 and *kailai*), valvae incurved at apex, costal margin sometimes with median bulge, sacculus widely crossing the valva with two (rarely three) processes on the posterior edge, the outer reaching close to the costal margin of valva or exceeding it, saccus often elongate, aedeagus slender, long with a single cornutus. Female genitalia with long ductus bursae. Species identification most often rests on the male genitalia, where the shape of the incurved apex of the valva, the length of the saccus and the relative proportions of the various parts provide diagnostic characters, in particular the length of the cornutus relative to the aedeagus and the length of the aedeagus relative to the length of the valva. Those species with known food-plants all feed on Apiaceae.

The presence of an undescribed species of this group in Spain was recognised by Klaus Sattler after he and David Carter collected several specimens in Sierra Nevada in 1969. These have remained unnamed in NHMUK since that date. Further specimens were later collected in the same area and elsewhere in Spain, most of these deposited in ZMUC. It was these that first came to the notice of M. Corley in 2004. Recently the species has been found in additional localities in Spain and in Greece. It is described here as *D. albarracinella* Corley sp. n.

The specimens from Greece have not been included in the type series. Although there is no reason to doubt the identification, the p-distance of over 1% between the barcodes of Spanish and Greek specimens suggests that caution is not out of place.

### Agonopterix carduncelli

Taxon classificationAnimaliaLepidopteraDepressariidae

Corley, sp. n. 

http://zoobank.org/F0BDBC85-90F3-41B9-8CE1-8DECC95F9CF8

#### Type locality.

Portugal, Algarve, Boliqueime, 70 m, 37°8'N; 8°1'W.

#### Holotype.

♂, **Portugal**, Algarve, Boliqueime, 24.xi.2011, M.J. Dale | *Agonopterix carduncelli* Corley Holotype | slide MD01355, DEEUR photo 0758 *A. carduncelli* | DNA barcode id. TLMF Lep 07015. Specimen to be deposited in NHMUK.

#### Paratypes.

**Portugal**: 1 ♂, Algarve, Boliqueime, 20.xi.2010, M.J. Dale, gen.prep. DEEUR 0757, in coll M.J. Dale; 1 ♂, Algarve, Mexilhoeira Grande, Cruzinha, 15.v.2011 ex l. iii.2011, Carthamus (Carduncellus) caeruleus, leg. M.F.V. Corley, DEEUR 0777, in coll. M. Corley; 1 ♀, same data but emerged 23.v.2011, gen. prep. DEEUR 0776, in coll. M. Corley; **Spain**: 1 ♂, Cuenca, Izotely, 30.ix.2008, leg. L. Srnka, gen. prep. DEEUR 2183, det. P. Buchner; **Greece**: 1 ♀, Messalongi Galatas, 5.v.2007, W. Schmitz, DEEUR 4404, det. P. Buchner; **Morocco**: 1 ♂, 1 ♀, High Atlas, Ifrane, 30.vi.1972, leg. F. Hahn, gen. prep. DEEUR 1983 (♂) bzw DEEUR 1980 (♀), det. P. Buchner; 1 ♂ same locality, 2.vii.1972, G. Friedel (ZSM), gen. prep. DEEUR 1677, det. P. Buchner.

#### Diagnosis.

The characteristic shape of segment 2 of the labial palp and the absence of a posterior crest on the thorax are features shared with a few other species mostly with similar coloration. *A. straminella* (Staudinger, 1870) is most similar with black dot at base of dorsum and black terminal dots together with paler hindwing, but lacks cell dots. Forms of *A. carduncelli* sp. n. without evident cell dots require genitalia examination to distinguish them from *A. straminella*. Other related species have better developed cell dots. In the male genitalia, *A. carduncelli* sp. n. is recognisable by the longer curved cuiller and broader valva in comparison with related species. The female is unique among European *Agonopterix* in the absence of a signum.

**Figures 8–9.**
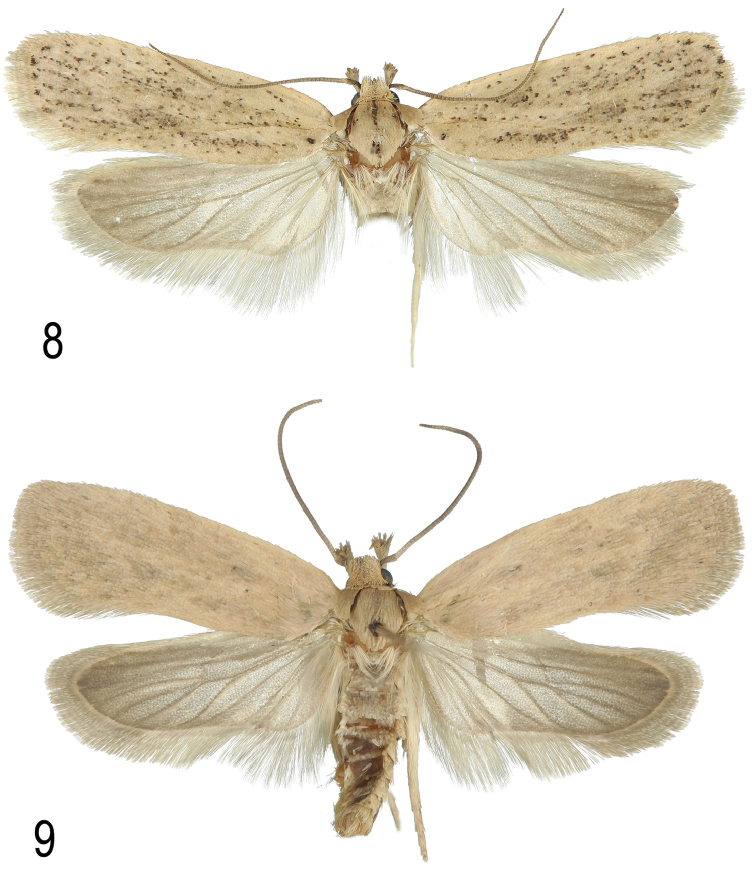
**8** *Agonopterix carduncelli* sp. n. Holotype male. Portugal, Algarve, Boliqueime, 24.xi.2011, leg. M.J. Dale **9** *Agonopterix carduncelli* sp. n. Paratype. Portugal, Algarve, Mexilhoeira Grande, Cruzinha, 15.v.2011, e.l. on *Carthamus* (*Carduncellus) caeruleus*, leg. M.F.V. Corley.

**Description. Adult** (Figs 8–9). Wingspan 19.5–21 mm. Head dull ochreous-buff, face creamy buff. Labial palp segment 2 with only the distal half rough-scaled and furrowed, pale buff with scattered light brown scales, segment 3 pale buff or ochreous-buff. Antenna with scape dull ochreous-brown, proximal part of flagellum ochreous-buff, ringed grey-brown, distally grey-brown. Thorax dull ochreous-buff often with darker median line, without posterior crest. Forewing pale ochreous-buff with faint pinkish tinge when fresh, with a variable amount of scattered light brown and blackish scales, particularly along veins towards termen and sometimes also in cell and between dorsum and fold; a black or brown dot at base of dorsum, a small dot in cell at two-fifths and usually another at end of cell; terminal spots dark grey-brown; a faint grey-brown stripe stretching through subdorsal area ending in a wider patch below end of cell; cilia pale ochreous-buff with weak cilia line. Hindwing light grey, darker in outer half; cilia light greyish ochreous with indistinct cilia lines. Legs pale ochreous-buff, foreleg blackish on upper side of tibia and part of tarsus. Abdomen light greyish buff.

Variation: Some specimens have many more scattered dark scales than others. The subdorsal spot can be distinct or dull pale brown; the cell dots may be obsolete, or if developed may still be indistinct due to the abundance of scattered scales; the development of the subdorsal streak is variable.

**Male genitalia.** (Fig. 12). Similar to related species, but gnathos almost exceeding socii by its own length, valva broader and smooth-sided, slightly curved, tapered to rounded apex; cuiller curving outwards at middle, parallel-sided, round and slightly wrinkled at apex, crossing four-fifths width of valva, longer than in related species due to broader valva. Fig. 13 shows male genitalia of the other six *pallorella* group species for comparison.

**Female genitalia.** (Fig. 14). Anterior margin of sternite VIII nearly straight, not bulging, ostium just beyond middle of plate; ductus bursae smooth, gradually expanding to corpus bursae; signum absent.

**Description of larva.** Head dark brown; prothoracic plate, thoracic legs and anal plate shining black; body deep purplish brown ; pinacula black. Full grown larva a little exceeding 20 mm.

**Figures 10–11.**
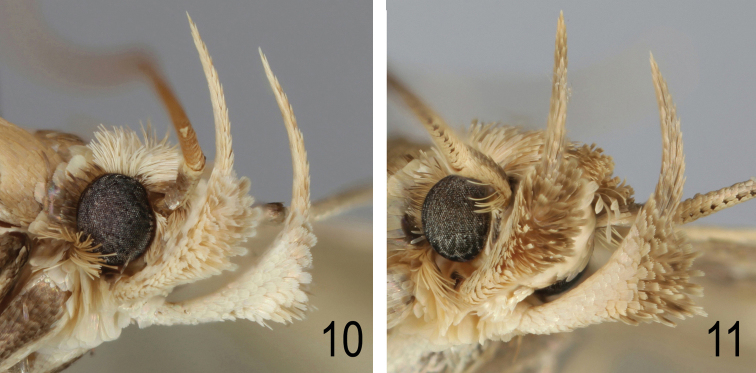
**10** *Agonopterix carduncelli* sp. n. Head. Portugal, Algarve, Boliqueime, 20.xi.2010, leg. M.J. Dale **11** *Agonopterix carduncelli* sp. n. Head. Mexilhoeira Grande, Cruzinha, 23.v.2011, e.l. on *Carthamus* (*Carduncellus) caeruleus*, leg. M.F.V. Corley.

**Figure 12.**
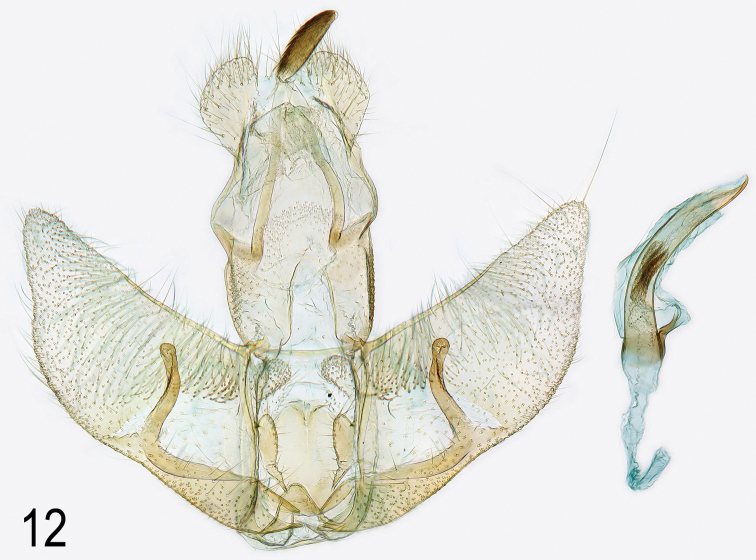
*Agonopterix carduncelli* sp. n. Male genitalia. Holotype. Portugal, Algarve, Boliqueime, 24.xi.2011, leg. M.J. Dale, slide MD01355 (gen. prep. and photo M. J. Dale).

**Figure 13.**
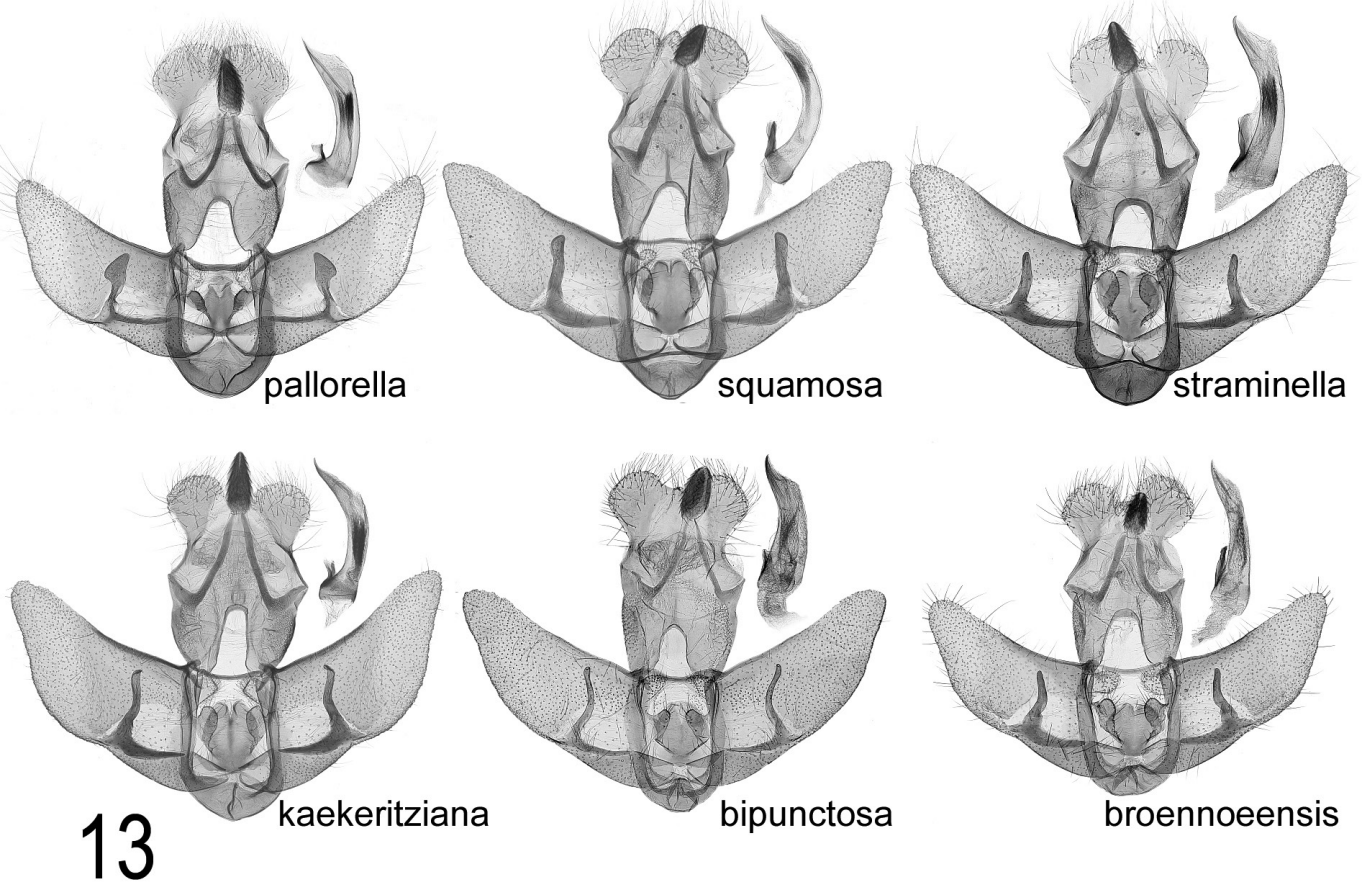
Comparison of male genitalia of European *A. pallorella* group species, excluding *A. carduncelli* sp. n. *A. pallorella* (Tunisia, Ksar); *A. squamosa* (Turkey, Amasia); *A. straminella* (Tunisia, Jebel Chambi); *A. kaekeritziana* (Austria, Schwarzau); *A. bipunctosa* (Sweden, Ronneby); *A. broennoeensis* (Russia, Kola, Apatity).

#### Molecular data.

**Data of barcoded specimens.** TLMF Lep 06978 (658 bp.[0n], ♂, Portugal, Mexilhoeira Grande, Cruzinha, 37°10'N; 8°37'W, leg. larva iii.2011 from *Carthamus* (*Carduncellus) caeruleus*, e.p. 23.v.2011, leg., cult. and coll. M. Corley P9827); TLMF Lep 06994 (620 bp.[0n], ♀, Portugal, Mexilhoeira Grande, Cruzinha, 37°10'N; 8°37'W, leg. larva iii.2011 from *Carthamus* (*Carduncellus) caeruleus*, e.p. 15.v.2011, leg., cult. and coll. M. Corley P9824, gen. prep. DEEUR 0776); TLMF Lep 07015 (658 bp.[0n], ♂, Holotype, Portugal, Algarve, Loulé, Boliqueime, 70 m, 37°8'N; 8°1'W, 24.xi.2011, gen. prep. MD01355, leg. and coll. M.J. Dale); TLMF Lep 07017 (658 bp.[0n], ♂, Portugal, Algarve, Boliqueime, 37°7'N; 8°9'W, 20.xi.2010, leg. and coll. M.J. Dale, gen. prep. DEEUR 0757).

**Neighbour-joining analysis** shows *Agonopterix multiplicella* (Erschoff, 1877) (BOLD:AAF7196, TLMF Lep 19102) as the nearest neighbour with 1.83% p-distance and *A. straminella* (BOLD:ABZ7581) as the second nearest neighbour with 2% p-distance. Intraspecific variability, based on present knowledge, 0.16% within the Portugese population. So far, genetic data are available only from Portugese specimens.

Differences in DNA barcodes arise over time through chance mutations. Such stochastic events sometimes lead to fairly unrelated species appearing as nearest neighbours. This is evidently the case with *A. carduncelli* sp. n. and *A. multiplicella*. The latter species has none of the characters of the *pallorella* group.

#### Etymology.

The species name, a noun in genitive case, is derived from the larval food-plant *Carthamus* [=*Carduncellus*] *caeruleus* (Asteraceae).

#### Distribution.

Currently known only from Portugal, Spain, Greece and Morocco, but potentially more widespread around the Mediterranean with its food-plant.

#### Bionomics.

The larva feeds in the tips of shoots of *Carthamus caeruleus* (L.) C. Presl in late March before the flowers develop. Larvae from Algarve collected on 17 March 2011 emerged in captivity in May. Small larvae were collected on 25 March 2017 (Portugal, Beira Litoral, Ansião, M. Corley and J. Nunes) and reared on by J. Nunes. Two reached the final instar (Figs 15–16) but succumbed to parasitoids.

**Figure 14.**
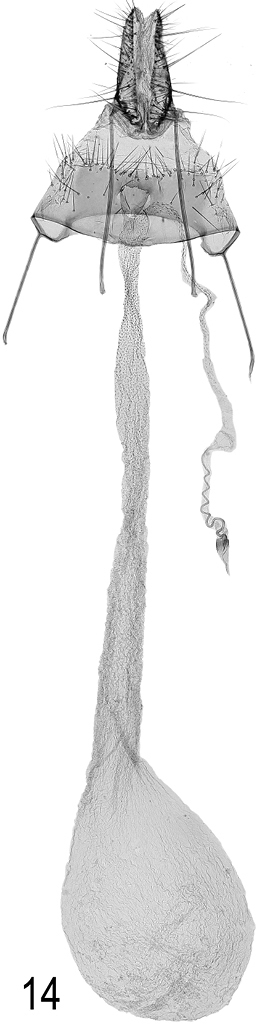
*Agonopterix carduncelli* sp. n. Female genitalia. Paratype. Morocco, Ifrane, 30.vi.1972, gen. prep. DEEUR 1980, leg. & coll. F. Hahn.

**Figure 15–16.**
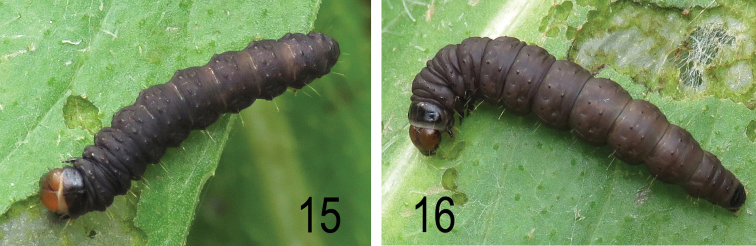
*Agonopterix carduncelli* sp. n Small larvae were collected on 25 March 2017 (Portugal, Beira Litoral, Ansião, M. Corley and J. Nunes) and reared on by J. Nunes. Two reached the final instar (Figs 15–16) but succumbed to parasitoids.

#### Remarks.

*Agonopterix* Hübner, 1825 with around 245 species (Wikipedia 2017) mainly in the Holarctic region is the largest genus in Depressariidae. Unlike *Depressaria*, male genitalia are rather similar throughout the genus. Easily defined groups within the genus are less obvious than in *Depressaria*, but there are some such groups. One of these is the *pallorella* group which includes several closely related species all feeding on Asteraceae tribe Cynareae and sharing similar pale ochreous coloration, forewings without defined basal patch, oblique pair of dots reduced to one or absent, labial palp characteristic with appressed scales on underside of segment 2 in proximal half, distal half with forward projecting scales making a triangular tuft, thorax without posterior crest. The species of this group are *A. pallorella* (Zeller, 1839), *A. kaekeritziana* (Linnaeus, 1767), *A. bipunctosa* (Curtis, 1850), *A. broennoeensis* Strand, 1920, *A. straminella* (Staudinger, 1870) and *A. squamosa* (Mann, 1864). A few other described taxa are synonyms of the above mentioned species. However one species has been previously overlooked and is described here as *A. carduncelli* sp. n.

The existence of an *Agonopterix* feeding on *Carthamnus* in Algarve, Portugal was suspected from the late 1990s when empty spinnings were found by M. Corley on the plant in late April. After Michael Dale found an adult of an undescribed species in 2010, a visit to Algarve in March 2011 by M. Corley targeting larvae on this plant was successful, resulting in two reared adults (see paratypes).

Two reared specimens (which survived the deterioration of their larval food-plant after M. Corley returned to England), show a grey-brown tinge in place of scattered dark scales and lack the row of terminal dots, but these features are shared by some of the Moroccan specimens.

Corley (2002) mentions a specimen without signum from Setúbal, Portugal in MNHN which was considered to be a possible aberration of *A. mendesi* Corley, 2002. As the signum is not known to be absent in any other *Agonopterix* species, it is extremely probable that this specimen belongs to *A. carduncelli* sp. n.

### Agonopterix pseudoferulae

Taxon classificationAnimaliaLepidopteraDepressariidae

Buchner & Junnilainen sp. n.

http://zoobank.org/8C70763C-9C9E-47CB-ABE7-F20B9271976C

#### Type locality.

Italy, Sardinia, Laconi, 39°51'N; 9°3'E.

#### Holotype.

♂, **Italy**, Sardinia, Laconi, 16.vi.2009, leg. J. Junnilainen, DNA barcode id. BC TLMF Lep 19306, gen. prep. DEEUR 4462, in coll. J. Junnilainen.

#### Paratypes.

1 ♀, same data as holotype, DNA barcode id. MM24152, leg. & coll. J. Junnilainen; 2 ♂♂, 3 ♀♀, same data as holotype, leg. & coll. J. Junnilainen; 1 ♂, 1 ♀, same data as holotype, leg. J. Junnilainen, in coll. ZMUH; 1 ♂, 1 ♀, same data as holotype, leg. J. Junnilainen, in coll. NHMUK; 1 ♂, 1 ♀, same data as holotype, leg. J. Junnilainen, in coll. M. Corley; 1 ♀, Greece, Peloponnese, Chelmos, 2100 m, 10.vii.1963, leg. J. Klimesch, coll. ZSM; 1 ♀, Italy, Puglia, 21.vii.1955, leg. S. Zangheri, in coll. ZSM; 1 ♂, Italy, Puglia, “FG“ Gargano sopra Vieste, 400 m, 5.ix.2008, coll. G. Fiumi; 1 ♂, Italy, Sicilia, Madonie, Piano Battaglia, 18.x.1990, gen. prep. DEEUR 1928, in coll. TLMF; 1 ♀, Italy, Latium, Mt Terminillo, 17.vii.2010, DNA barcode id. TLMF Lep 19067, gen. prep. DEEUR 1737, leg. & coll. T. Mayr; 1 ♂, 1 ♀, Italy, Puglia, Gargano, leg. larva 4.iv.2016 from *Elaeoselinum asclepium*, e.p. end of April 2016, leg., cult. & coll. P. Sonderegger.

**Diagnosis.** *A. pseudoferulae* sp. n. (Figs 19–27) was confused with *A. ferulae* (Figs 28–29) by Klimesch. At first glance, they do look similar, but a closer look shows two constant differences: the brick-red line between the proximal pair of dots and the distal dot and the diffuse dark spot which touches this line on costal side in *A. pseudoferulae* sp. n., which are both absent in *A. ferulae* (if diffuse dark spots are present, they are found in other areas). *A. atomella* (Denis & Schiffermüller, 1775) (Fig. 33) and *A. scopariella* (Heinemann, 1870) (Fig. 32), the two species closest to *A. pseudoferulae* sp. n. in DNA barcode, do not have the reddish elements in central forewing pattern. *A. oinochroa* (Turati, 1879) (Fig. 31) has reddish elements here, but they surround the dots and do not form a line. A forewing pattern similar to *A. pseudoferulae* sp. n. is found only in *A. cluniana* Huemer & Lvovsky, 2000 (Fig. 30), but here differences are found in outline of forewing and shape of interneural dots at outer margin: apex rounded, outer margin convex, interneural dots round and diffuse in *A. pseudoferulae* sp. n., apex pointed, outer margin straight to concave, interneural dots narrow lines in *A. cluniana*.

#### Description.

**Adult**: Wingspan 19–21 mm. Scales of head brown, tips markedly paler. Labial palp segment 2 inner side pale, outer and ventral sides medium greyish brown or rusty brown scales mixed with blackish scales; third segment bicoloured, blackish at base, shortly above middle and at extreme tip, pale between the dark areas. Antenna dark brown. Thorax with posterior crest, rather dark brown, tegulae similar. Forewing predominantly dark reddish brown, whitish and black scales interspersed in low (but variable) numbers, basal field markedly paler, gradually passing into a pale stripe which runs along costa especially in proximal half and is interrupted by irregular dark patches. The centre of the forewing has the typical basic pattern of *Agonopterix* (two oblique dots at about one-third, one or two dots along veins at about one-half and a diffuse black spot between the two pairs of dots but closer to the costa) but with very distinct details: the two black, oblique dots partly bordered with reddish (brick-red to ochreous) scales which may connect the two dots on their proximal margin, distal margin pale to white, the third dot at about one-half with clear white centre and surrounded by a few dark scales, a brick-red to ochreous line connects the oblique dots with the distal dot and exeeds it a little; the diffuse blackish spot touches the brick-red line between the dots on the costal side. Cilia concolorous with wings. Under side of forewing dark grey except costa which is predominantly yellowish with interspersed groups of dark scales. Hindwing rather dark greyish brown, moderately translucent at base, cilia concolorous with wings, base and tips darker than in between. Legs covered with a mix of dark grey and pale scales, tibia yellowish to rusty brown on outer side, especially on fore- and hindlegs. Abdomen greyish, with broad dark line laterally and two rows of indistinct dark spots on ventral side.

No gender-specific differences could be found.

Variation: Little variation was found within the nine examined specimens. The number of interspersed white scales on forewing varies to some extent, and between the proximal pair of dots and the distal dot, an additional white dot may be developed or not. In one specimen the thorax (but not tegulae) is entirely black.

**Male genitalia** (Fig. 34): There is no single feature which separates male genitalia of *A. pseudoferulae* sp. n. from all other species of *Agonopterix*, therefore it is best to compare genitalia with each of the externally similar species individually.

*A. ferulae* (Fig. 35) belongs to the *alpigena/selini* species group, which is characterised by a two-horned process of the anellus toward transtilla (see arrow in insert of Fig. 35) in combination with transtilla significantly widened in the middle. In *A. pseudoferulae* sp. n. these features are not present. In *A. atomella* (Fig. 36), *A. oinochroa* (Fig. 37) and *A. scopariella* (Fig. 38) anellus lobes are large, nearly touching in *A. atomella* and *A. oinochroa*, overlapping in *A. scopariella* in standard preparation. In *A. pseudoferulae* sp. n. anellus lobes are narrow with a wide gap in between. In *A. cluniana* (Fig. 39) cuiller is rather short (about 70% of valva-width) and socii markedly narrow, gnathos far exceeding end of socii, in *A. pseudoferulae* sp. n. cuiller nearly reaching costa of valva (at least 90% of valva-width), socii and gnathos of average shape, compare Figs 34 and 39 (appearance of socii and gnathos may be influenced by preparation artifacts, so it is not helpful to point out a numerical ratio).

**Female genitalia** (Figs 40, 41a). Anterior margin of sternite VIII with a triangular process, which is separated from lateral parts of anterior margin by distinct steps (arrows 41a), ostium round, in the centre of sternite VIII, not reaching into the triangular process. Ductus seminalis with about 8 turns. Ductus bursae rather stout with structures common in genus *Agonopterix*, widening gradually in its course. Corpus bursae of average size (diameter approximately equalling width of sternite VIII in standard preparation, i.e. dorsoventrally flattened), signum narrow oval (4 times wider than long), rather large (maximum diameter about one half diameter of bursa).

As in the males, all the species compared with *A. pseudoferulae* sp. n. also show distinct differences in female genitalia: *A. ferulae* (Fig. 41b) has a straight margin of sternite VIII without any fold. In *A. atomella*, oblique folds (arrow Fig. 41c) are developed at each side of centre of margin. In *A. scopariella* these folds are also present and between them, the anterior margin shows a somewhat rectangular extension (arrow Fig. 41d). In *A. oinochroa* it is slightly curved with a narrow transverse fold (arrow Fig. 41e), in *A. cluniana* it is extremely bulged (arrow Fig. 41f), which gives a character of the female genitalia of this species which is unique within *Agonopterix*.

**Figure 17.**
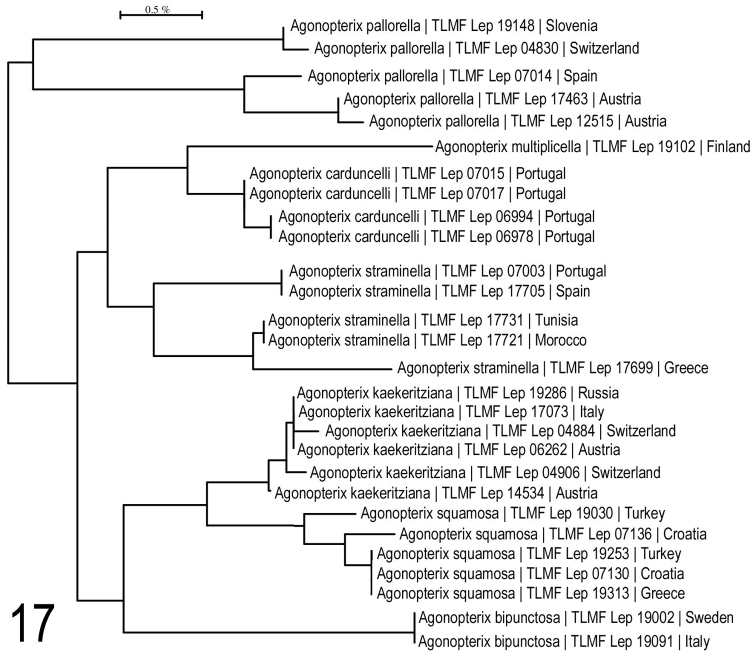
Neighbour-joining tree of species of *pallorella*-group and *A. multiplicella*. Associated BOLD BINs: *A. pallorella*: BOLD:ABA0382 (upper cluster); BOLD:ABU5790 (lower cluster); *A. multiplicella*: BOLD:AAF7196; *A. carduncelli*: BOLD:ABZ7583; *A. straminella*: BOLD:ABZ7581 (upper cluster) BOLD:ACX7863 (lower cluster); *A. kaekeritziana*: BOLD:AAF7198; *A. squamosa*: BOLD:ACF7120; *A. bipunctosa*: BOLD:ABA0011.

**Figure 18.**
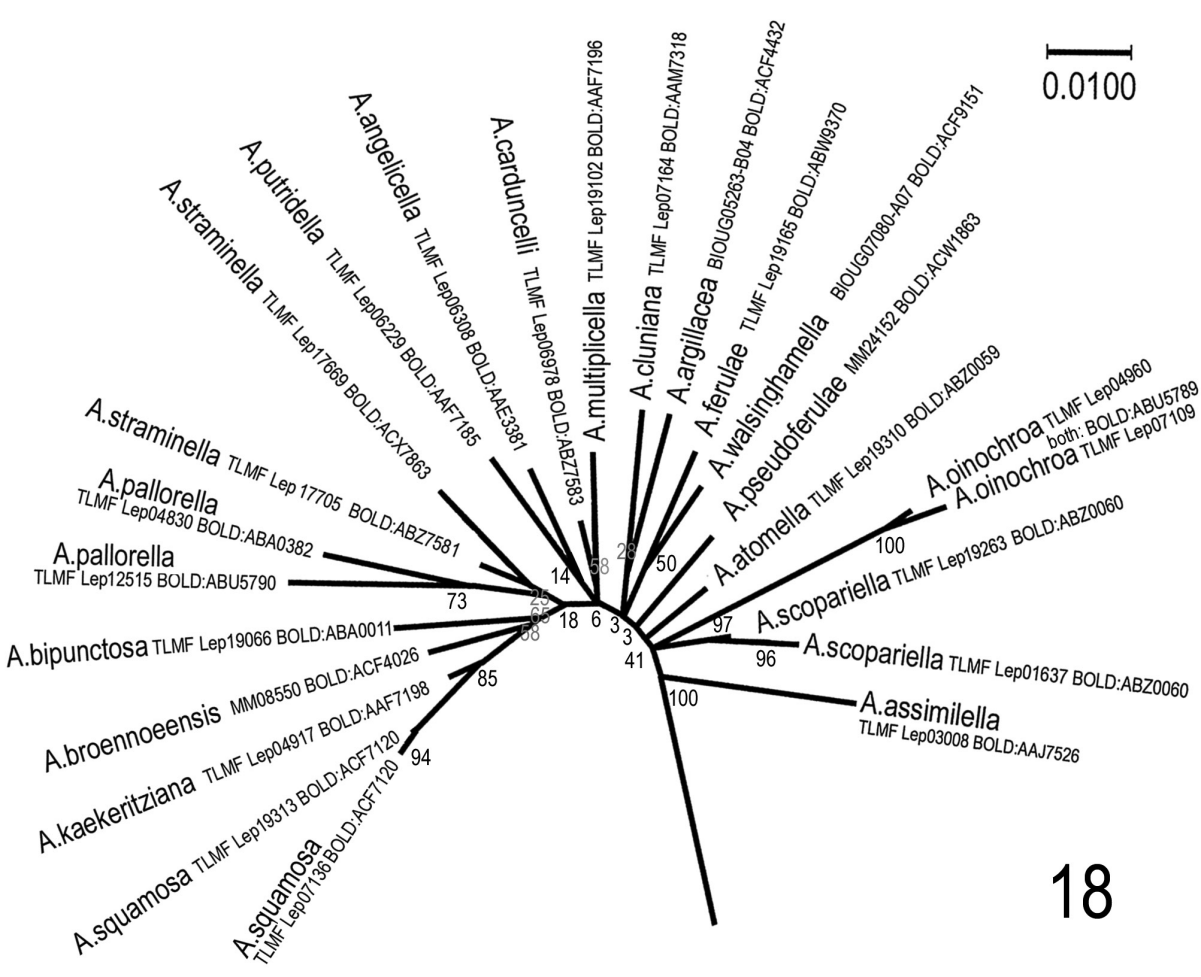
Maximum Likelihood analysis of selected species of the genus *Agonopterix*: *A. pallorella*-group, *A. multiplicella* and species near *A. pseudoferulae*. In addition to the selection used for the neighbour-joining tree, the following *Agonopterix* species were included: *A. angelicella* (Hübner, [1813]) (TLMF Lep 06308, BOLD:AAE3381); *A. argillacea* (Walsingham, 1881) (BIOUG05263-B04, BOLD:ACF4423); *A. assimilella* (Treitschke, 1832) (TLMF Lep 03008, BOLD:AAJ7526); *A. walsinghamella* (Busck, 1902) (BIOUG07080-A07, BOLD:ACF9151). The evolutionary history was inferred by using the Maximum Likelihood method based on the Tamura-Nei model. The tree with the highest log likelihood (-4818.52) is shown. Initial trees for the heuristic search were obtained automatically by applying Neighbor-Join and BioNJ algorithms to a matrix of pairwise distances estimated using the Maximum Composite Likelihood (MCL) approach, and then selecting the topology with superior log likelihood value. The tree is drawn to scale, with branch lengths measured in the number of substitutions per site. The proportion of sites where at least 1 unambiguous base is present in at least 1 sequence for each descendent clade is shown next to each internal node in the tree. The analysis involved 54 nucleotide sequences from selected species of *Agonopterix* and *Depressaria*. Only the *Agonopterix* part of the tree is shown here. Codon positions included were 1st+2nd+3rd+Noncoding. All positions containing gaps and missing data were eliminated. There were a total of 657 positions in the final dataset. Evolutionary analyses were conducted in MEGA7: Molecular Evolutionary Genetics Analysis version 7.0 for bigger datasets (Kumar, Stecher and Tamura 2015). The result is shown in radiation graphic, because in this view the evolutionary aspect is visualized better than in rectangular tree.

**Figures 19–22.**
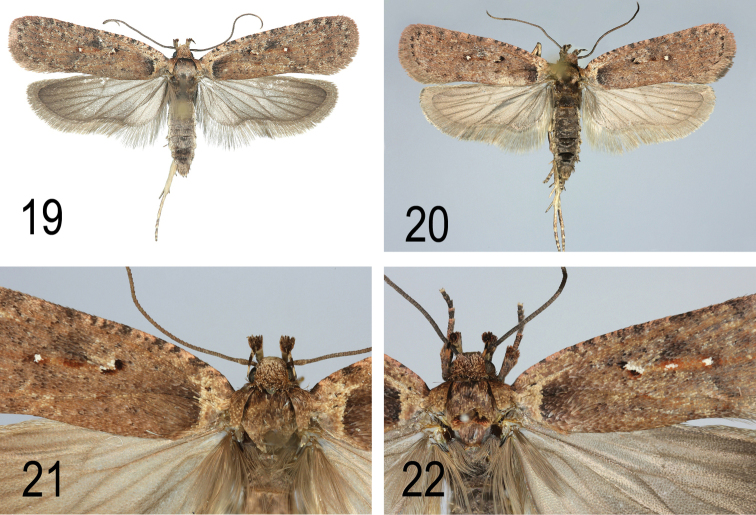
**19** *A. pseudoferulae* sp. n. Holotype (Italy, Sardinia, Laconi), general view **20** *A. pseudoferulae* sp. n. Paratype. (Italy, Sardinia, Laconi), general view **21** *A. pseudoferulae* sp. n. Paratype. (Greece, Peloponnese), head, thorax and forewing base **22** *A. pseudoferulae* sp. n. Paratype. (Italy, Puglia, Gargano, e.l. *Elaeoselinum asclepium*), head, thorax and forewing base.

**Figures 23–27.**
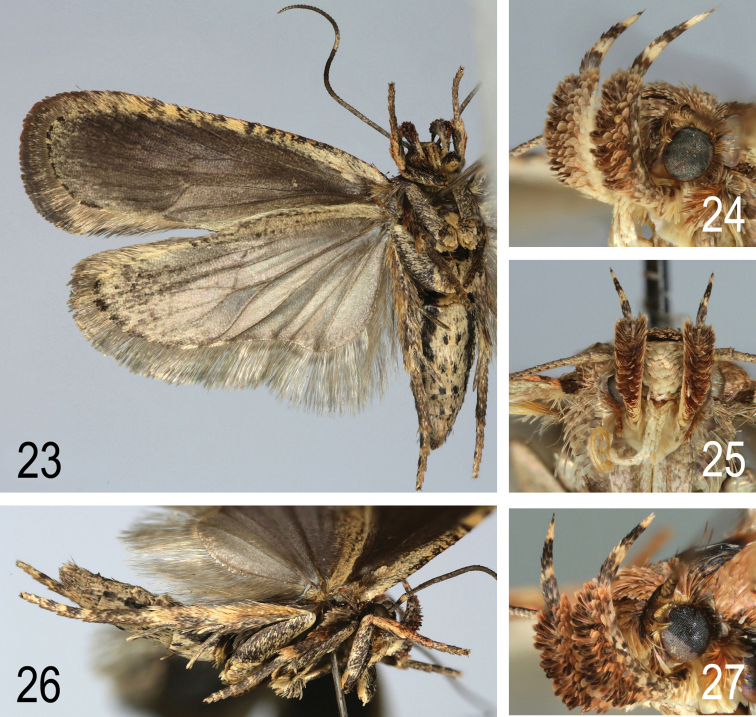
**23** *A. pseudoferulae* sp. n. Paratype. (Italy, Gargano, e.l. *Elaeoselinum asclepium*), ventral view **24–25** *A. pseudoferulae* sp. n. Paratype (Greece, Peloponnese), palps (**24** lateral view **25** frontal view) **26** *A. pseudoferulae* sp. n. Paratype (Italy, Gargano, e.l. *Elaeoselinum asclepium*), lateral view **27** *A. pseudoferulae* sp. n. Paratype (Italy, Mt Terminillo), palps, lateral view.

**Figures 28–33.**
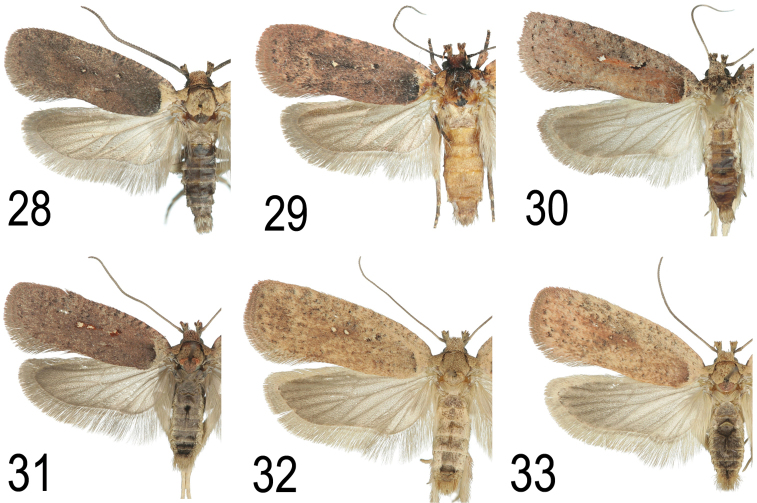
Comparison of wing patterns of several species similar to *A. pseudoferulae* sp. n. **28** *A. ferulae* (France, Var, e.l. *Ferula communis*) **29** *A. ferulae* (Italy, Sardinia, Gennargentu) **30** *A. cluniana* (Austria, Vorarlberg, Bangs) **31** *A. oinochroa* (Germany, Kaiserstuhl, e.l. *Genista tinctoria*) **32** *A. scopariella* (Italy, Lugano, e.l. *Laburnum*) **33** *A. atomella* (Austria, Lower Austria, Waschberg).

**Figures 34–39.**
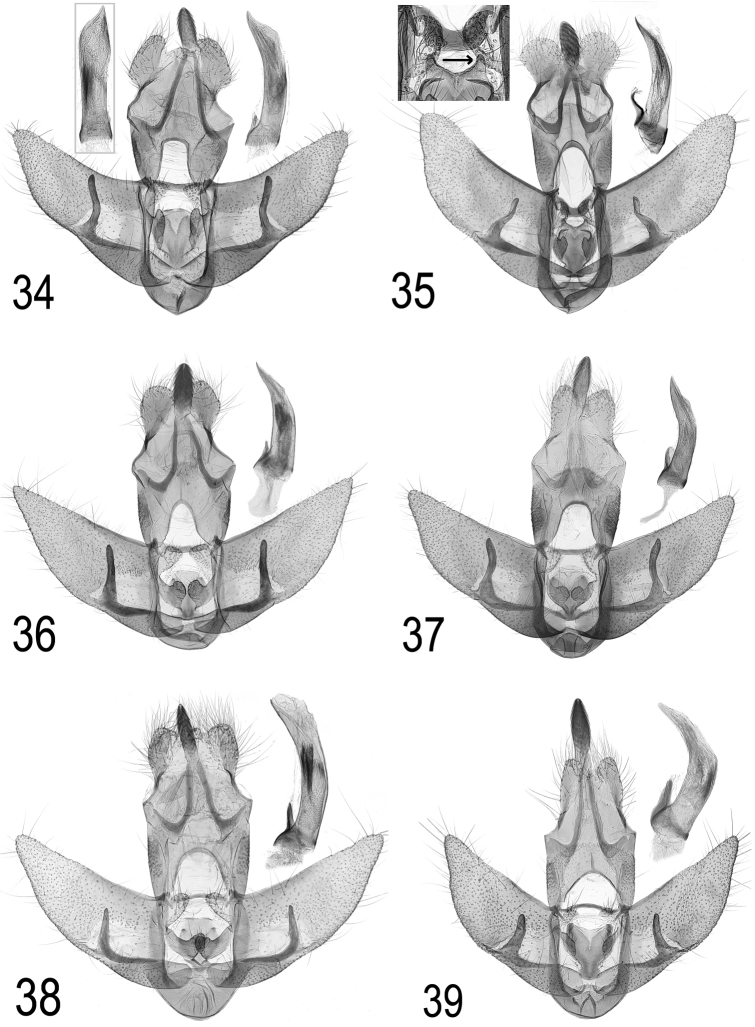
Comparison of male genitalia of *A. pseudoferulae* sp. n. with selected species. **34** *A. pseudoferulae* sp. n. Holotype, insert: aedeagus in ventral view **35** *A. ferulae* (Portugal, Trás-os-Montes), insert: anellus process and transtilla **36** *A. atomella* (Italy, Friuli, Redipuglia) **37** *A. oinochroa* (Spain, Leon, Vilafeliz de Babla) **38** *A. scopariella* (Croatia, Novi Vinodolsky) **39** *A. cluniana* (Austria, Vorarlberg, Bangs).

**Figures 40–41.**
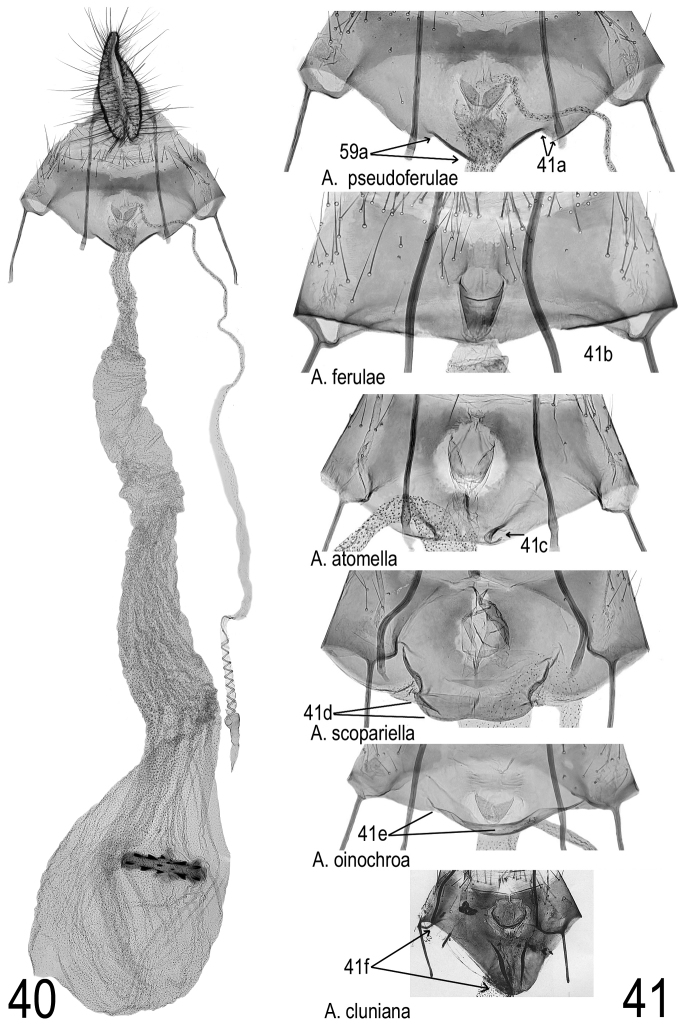
Comparison of female genitalia of *A. pseudoferulae* sp. n. with selected species. **40** *A. pseudoferulae* sp. n., general view, paratype (Italy, Latium, Mt Terminillo) **41** ostium region of six selected species enlarged, arrows: see text under “female genitalia” **41a** *A. pseudoferulae* sp. n. (same specimen as Fig. 40) **41b** *A. ferulae* (Italy, Sardinia, Gennargentu) **41c** *A. atomella* (Austria, Lower Austria, Waschberg) **41d** *A. scopariella* (Portugal, Madeira, Faja da Nogueira) **41e** *A. oinochroa* (Serbia, Deliblato Sands) **41f** *A. cluniana* (Austria, Vorarlberg, photo from Huemer & Lvovsky, 2000).

#### Molecular data.

**Data of barcoded specimens**: BC TLMF Lep 19306 (658 bp., holotype, ♂, Italy, Sardinia, Laconi, 39°51'N; 9°3'E, 16.vi.2009, leg. & coll. J. Junnilainen); MM24152 (658 bp., ♀, same locality as holotype, leg. & coll. J. Junnilainen); TLMF Lep 19067 (658 bp., ♀, Italy, Latium, Mt Terminillo, 1600m, 42°29'N; 13°0'E, 17.vii.2010, gen. prep. DEEUR 1737 P. Buchner, leg. & coll. T. Mayr).

**Neighbour-joining analysis** shows *Agonopterix atomella* ([Denis & Schiffermüller], 1775) (BOLD:ABZ0059) as the nearest neighbour at a minimum of 2.45% p-distance. So far there are only sequences from the Italian population available, where no intraspecific divergence had been found, but this may change when Greek specimens are sequenced.

For Maximum Likelihood analysis, see Fig. 18.

**Figure 42.**
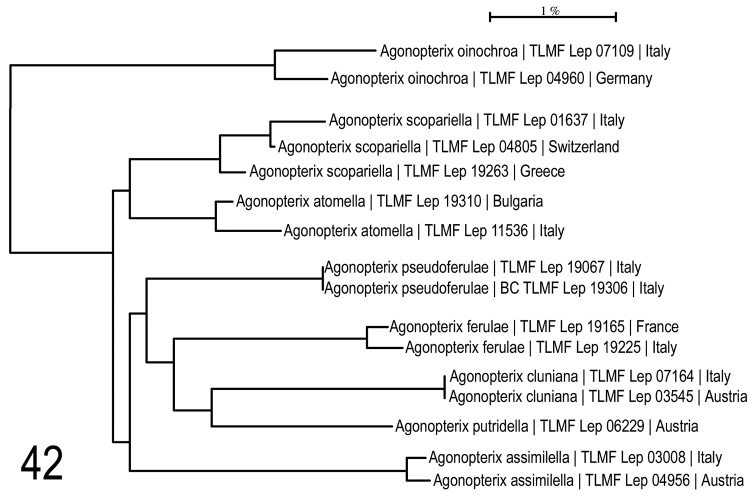
Neighbour-joining tree of *Agonopterix pseudoferulae* sp. n. and its closest clusters. Associated BOLD BINs: *A. oinochroa*: BOLD:ABU5789; *A. scopariella*: BOLD:ABZ0060; *A. atomella*: BOLD:ABZ0059; *A. pseudoferulae*: BOLD:ACW1863; *A. ferulae*: BOLD:ABW9370; *A. cluniana*: BOLD:AAM7318; *A. putridella*: BOLD:AAF7185; *A. assimilella*: BOLD:AAJ7526.

**Related species**: Searching for the most closely related species based on a neighbour-joining tree (Fig. 42), Maximum Likelihood analysis (Fig. 18) and genitalia patterns of both sexes has not achieved a satisfactory result in *A. pseudoferulae*. Compared with the nearest neighbour, there are some distinct differences: *A. atomella* is a Fabaceae-feeder, in male genitalia anellus lobes are very different. Looking further afield at the second nearest neighbour, *Agonopterix scopariella* (Heinemann, 1870) with a p-distance of 2.6% is also a Fabaceae-feeder, and the differences in genitalia are at least as marked as in *A. atomella*. On the other hand, the two Fabaceae-feeders *A. atomella* and *A. oinochroa* have very similar male genitalia, but a barcode distance of 4.08%. This suggests that every single parameter must be handled with care. Pronounced similarity may result from being closely related, but it does not prove it, because a single distinctive feature may develop independently in different groups. The only certainty from present evidence is that *A. pseudoferulae* is not a cryptic species.

#### Etymology.

The species name is a noun in genitive case. The first specimens of this new species were discovered in ZSM in the Klimesch collection under *A. ferulae*. This was decisive for the species name *pseudoferulae*, which means “the false *ferulae*”.

#### Distribution.

So far known from Italy and Greece. In Italy it had been collected from Mt Terminillo (Latium), Gargano (Puglia), Madonie, Piano Battaglia (Sicily) and Laconi (Sardinia) and in Greece from Chelmos (Peloponnese).

#### Bionomics.

Peter Sonderegger reared it from larvae collected on *Elaeoselinum asclepium* (L.) Bertol. (Apiaceae) from Gargano, Italy. Unfortunately he was not expecting anything of great interest, so no photo or larval description was obtained. Larvae were collected on 4 April, while the moth emerged in late April. Moths in good condition have been caught in June and July, and a worn specimen has been caught in October. It remains unclear in which stage the species survives winter.

#### Remarks.

In ZSM under the name *Agonopterix ferulae* P. Buchner found a specimen collected by Josef Klimesch in Greece, which had a red mark in the discal cell which is not present in *A. ferulae*. Genitalia examination showed that it was distinct from *A. ferulae* and subsequently, when more recent specimens were found it was possible to obtain barcodes. Both genitalia and barcodes show this to be a new species not closely related to *A. ferulae*. It is described here as *A. pseudoferulae*.

### Depressaria saharae Gaston & Vives ssp. tabelli

Taxon classificationAnimaliaLepidopteraDepressariidae

Buchner ssp. n.

http://zoobank.org/C90466AD-E3A7-4BF8-A452-ECFDD33E93EF

#### Type locality.

Spain, Canary Islands, Tenerife, Guimar.

#### Holotype.

♂, Spain, Canary Islands, Tenerife, Guimar, 6.iii. *Bupleurum aciphyllum* [Bupleurum salicifolium ssp. aciphyllum], ex. 16.iv.1907, Wlsm. 99748 | Walsingham Collection 1910-427 | B.M. ♂ Genitalia Slide No. 23304, NHMUK010305296, coll. NHMUK.

#### Paratypes.

1 ♀, Spain, Canary Islands, Tenerife, Guimar, La Ladera, 800 m, 23.iv.1998, GP DEEUR 2634, DNA-barcode id TLMF Lep 17692 (658 bp., BOLD:ADC8281), leg. & coll. K. Larsen,; 1 ♀, Tenerife, Los Gigantes, 100 m, 8-11.i.2008, GP DEEUR 2807, DNA-barcode id TLMF Lep 17711 (658 bp., BOLD:ADC8281), leg. & coll. K. Larsen.

#### Other material examined.

***Depressaria saharae ssp. saharae*** . 1 ♂, Spain, Granada, Sierra Nevada, 2430 m, 37°6.23'N; 3°23.84'W, 3.vii.2015, J. Tabell leg., GP ♂ 5480 J. Tabell, DEEUR 4024, DNA barcode id. TLMF Lep 19164 (658 bp., BOLD:ACF8051); 1 ♂, same collection data, without barcode; 2 ♂♂, Teruel, Albarracin, Val de Vecar, 1100 m, 3.x.2015, leg. J. Viehmann, coll. W. Schmitz; 1 ♂, Sr. de Albarracin, Sr. Alta, 1750m, 25.vi.2016, leg. J. Viehmann, coll. W. Schmitz; 3 ♂♂, Teruel, Albarracin. 6 km env. 1.x.2008, GP DEEUR 1000 & 1005, leg. & coll. L. Srnka, 1 of them (GP DEEUR 1000) with DNA barcode id. TLMF Lep 07068 (584 bp., BOLD:ACF8051)

**Introductory note**. It may be considered unusual to give a detailed description of the nominate subspecies before the description of a new subspecies, but in this case the original Spanish description is not detailed enough to serve as the basis for a comparison of the two subspecies. The original description is completely without information on genetic data and has little on relationships of the new species.It is therefore necessary to include such information on the nominate subspecies in this investigation.

#### Diagnosis.

The wing pattern of both subspecies of *D. saharae* belongs to one of the basic patterns in the genus *Depressaria* which can also be found e.g. in *D. ultimella* Stainton, 1849 and *D. daucella* (Denis & Schiffermüller, 1775), with which this species was confused by Walsingham (published by him as *Depressaria apiella* (Hübner, 1796)). A situation which is often found in *Depressaria* is a combination of high intraspecific variability and near identical basic wing patterns used by several species, which makes it very difficult to determine specimens externally. When intraspecific variability is larger than the mean difference between the species, identification may become impossible. On the other hand, most species of *Depressaria* have distinctive genitalia in both sexes. This is the case in *D. saharae*, where diagnosis must be based on genitalia: see relevant paragraphs below.

#### Description.

**Figure 43.**
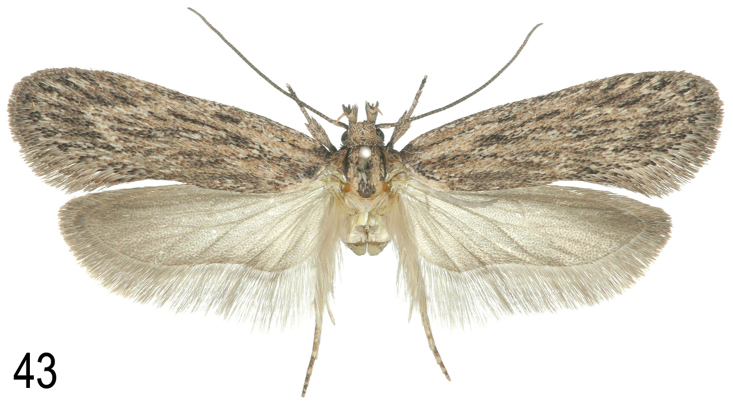
Depressaria saharae ssp. saharae Spain, Granada, Sierra Nevada, 3.vii.2015, J. Tabell leg, general view.

**Figures 44–47.**
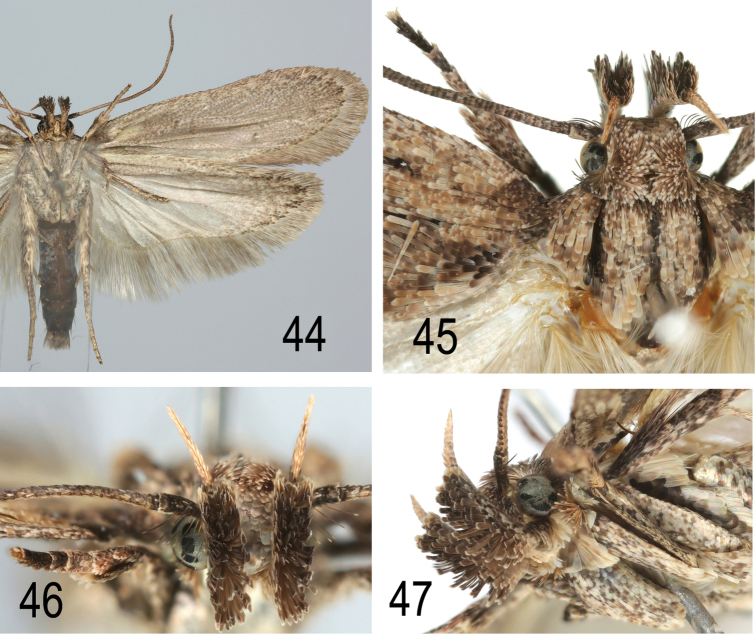
Depressaria saharae ssp. saharae same data as Fig. 43, but another specimen **44** lower side **45** head and thorax **46** labial palp, frontal view **47** labial palp, lateral view.

Depressaria saharae ssp. saharae specimens (only males) from mainland Spain (Figs 43–47): Wingspan 18–23 mm. Head greyish brown, tips of the scales markedly paler than the rest. Labial palp second segment with long, forward projecting scales which are dark grey with a narrow whitish distal margin, third segment medium grey with flesh-coloured tinge, only at base with some blackish scales. Antenna with scape blackish, flagellum blackish on dorsal side and medium yellowish grey on ventral side. Thorax and tegulae medium greyish brown, thorax with 3 dark longitudinal streaks, one in the middle and one at each side. Forewing ground colour grey, with distinct blackish longitudinal streaks, especially in outer one-third; whitish scales are interspersed in low numbers over the whole surface, also forming an acute angled transverse line at about two-thirds, angle about 50°, and a longitudinal, somewhat interrupted line in the middle from about one-fifth to one-half; in older specimens the patterns formed by the whitish scales soon become invisible, but the longitudinal blackish streaks remain visible even in rather worn specimens; cilia dark grey, without distinct contrast from wings. Hindwing moderately translucent at base, becoming increasingly opaque toward distal part, medium greyish brown, veins darker; cilia concolorous with wings, basal one-third markedly darker than the rest in fresh specimens. Legs and abdomen without distinct patterns, covered with a mixture of light grey and blackish scales.

**Figures 48–53.**
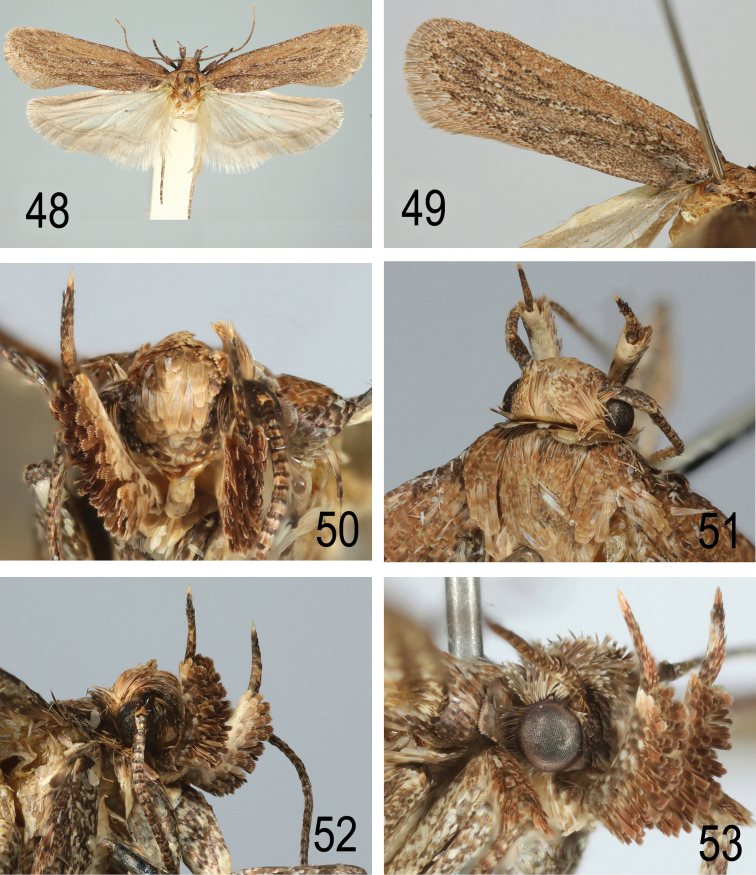
**48** *Depressaria saharae ssp. tabelli* ssp. n., holotype, Spain, Canary Islands, Guimar, e.l. *Bupleurum aciphyllum* 16.iv.1907 **49** Depressaria saharae ssp. tabelli ssp. n., Spain, Canary Islands, Guimar, 23.iv.1998, left forewing and palp **50–52** same specimen, details of head and palp **53** *Depressaria bupleurella*, Austria, Mannersdorf, 21. iii. 2016, leg. W. Stark.

Depressaria saharae ssp. tabelli ssp. n. (Figs 48–52): Wingspan 22–24 mm. Head warm yellowish brown, tips of the scales only slightly paler than the rest. Labial palp second segment with long, forward projecting scales which are dark warm brown with a narrow whitish distal margin, third segment yellowish at the very tip, rest of distal half predominatly black, basal half with varying proportions of blackish and pale scales. Antenna as in nominate ssp. Thorax and tegulae warm medium brown, thorax without black longitudinal streaks, only a slightly darker shadow may be visible. The most striking differences are colour and patterns of forewings: ground colour warm medium brown in costal half, becoming darker in dorsal half, but without sharp borderline between these areas, longitudinal streaks reduced, much less prominent than in nominate ssp., in central part of costal half almost completely absent; interspersed whitish scales and acute angled transverse line as in nominate ssp., cilia following the general tendency more warm brown, no remarkable difference in hindwings, legs and abdomen.

No gender-associated differences could be found in the specimens from Canary Islands.

**Figures 54–57.**
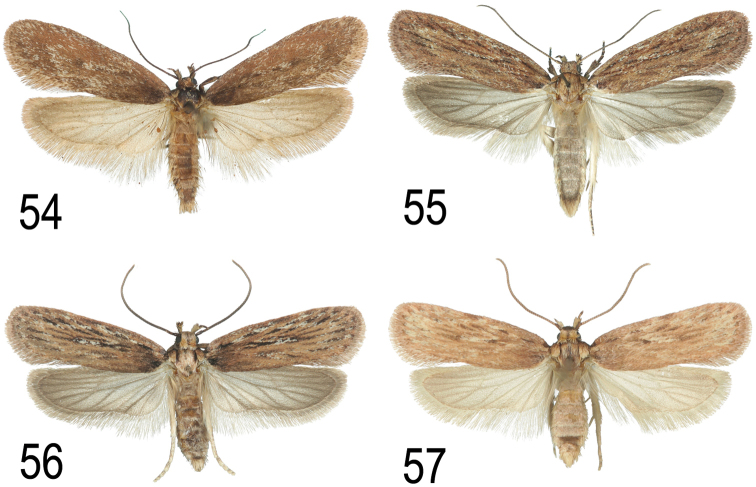
**54** *Depressaria bupleurella*, Germany, Pfalz, leg. Eppelsheim 1893, coll. NHMV **55** *Depressaria daucella*, Austria, Perchtoldsdorf, leg. P. Buchner 2012 **56** *Depressaria ultimella*, Sweden, Öland, e.l. *Cicuta virosa*, leg. R. Johansson 1990, coll. ZMUC **57** *Depressaria ultimella*, Belgium, Frameries, e.l. *Apium graveolens*, leg. A. Dufrane 1935, coll. ZSM.

For comparison, *D. bupleurella* (Fig. 54), *D. daucella* (Fig. 55) and two forms of *D. ultimella* (Figs 56–57) are shown.

**Figures 58–61.**
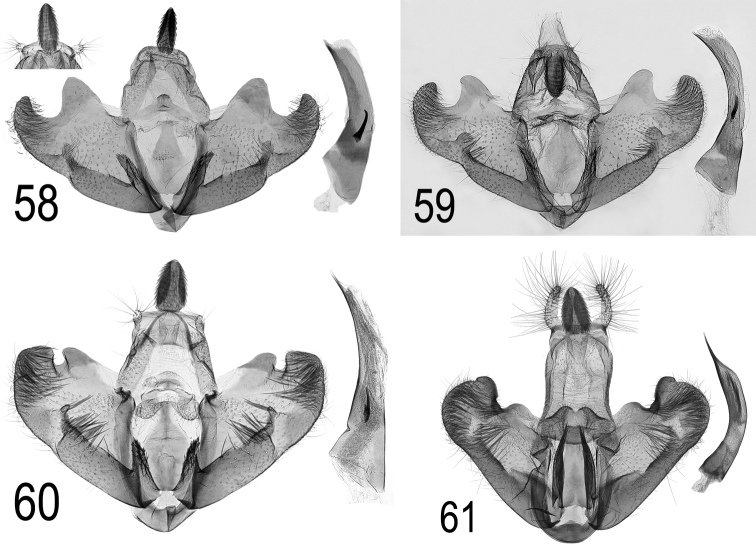
**58** Depressaria saharae ssp. saharae, preparation Jukka Tabell, slide 5480 J. Tabell; insert top left: D. saharae ssp. saharae, Spain, Teruel, 1.x.2008, with clearly visible socii, DEEUR 1000 **59** *D. saharae ssp. tabelli* ssp. n. holotype, Canary Islands, 16.iv.1907, coll. NHMUK, preparation Klaus Sattler, B.M. genitalia slide 23304 **60** *D. bupleurella*, Austria, Klosterneuburg, e.l. *Bupleurum*, 1922, coll. NHMV, slide MV18258 **61** *D. radiella*, Russia, Caucasus, leg. L. Srnka 2013, slide DEEUR 2174.

**Male genitalia.** Male genitalia of *D. saharae* (Figs 58–59) are really similar only to those of *D. bupleurella* Heinemann, 1870. The most distinctive difference is the width of the excavation in the costa of valva: narrow (less than half of the basal diameter of the bulges at each side of the excavation) in *D. bupleurella* (Fig. 60), wide (about equalling the basal diameter of these bulges) in *D. saharae*. Apart from the species pair *D. bupleurella / saharae*, the genitalia of *D. radiella* (Goeze, 1789) show some similarity, but with differences in many details; see comparison in Figs 58–61.

**Figures 62–68.**
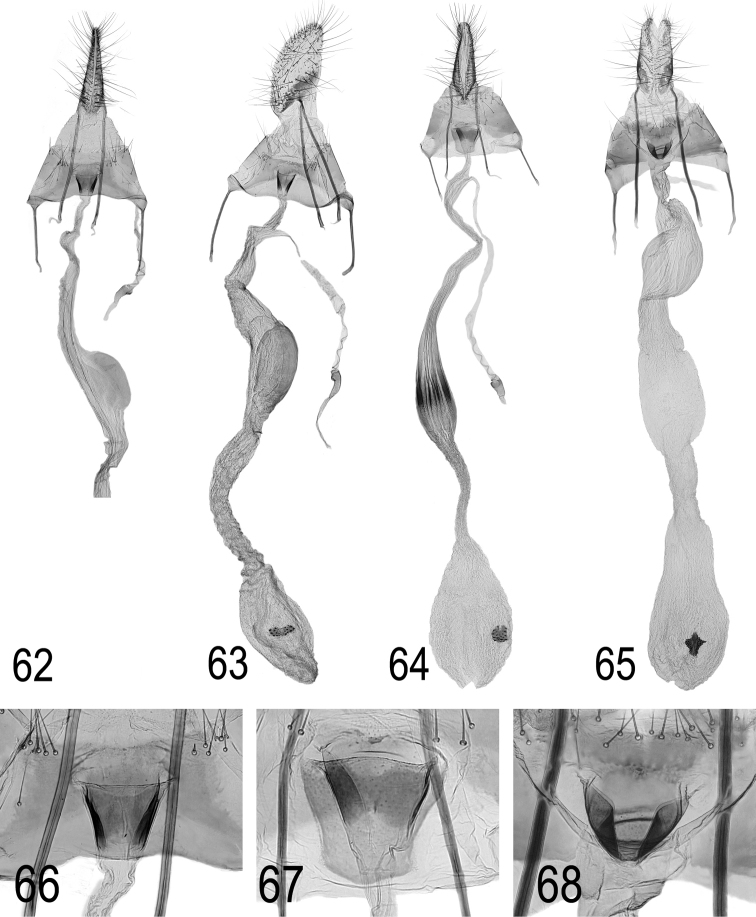
**62 + 66** Depressaria saharae ssp. tabelli ssp. n. Spain, Canary Islands, Los Gigantes, 11.i.2008, slide DEEUR 2807 (**62** general view **66** ostium region enlarged) **63** *D. saharae ssp. tabelli* ssp. n. Spain, Canary Islands, Guimar, 23.iv.1998, slide DEEUR 2634 **64 + 27** *D. bupleurella*, Italy, coll. TLMF, slide DEEUR 1646 (**64** general view **67** ostium region enlarged) **65 + 68** *D. radiella*, Austria, leg. & coll. P. Buchner, slide DEEUR 0029 (**65** general view; **68** ostium region enlarged).

**Female genitalia.** Female genitalia (Figs 62–63 + 66) are also most similar to *D. bupleurella* (Figs 64 + 67) with nearly the same shape of ostium and an expansion in the middle of the long and narrow ductus bursae. The best feature to separate the species is the shape of the expansion: an asymmetrical swelling without longitudinal streaks in *D. saharae ssp. tabelli* but spindle-shaped with several longitudinal sclerotisations in *D. bupleurella. D. radiella* is also figured for comparison (Figs 65 + 68). Females of the nominate ssp. are unknown so far.

#### Molecular data.

**Data of barcoded specimens.** TLMF Lep 19164 (658 bp., ♂, Spain, Granada, Sierra Nevada, 2430 m, 37°6.23'N; 3°23.84'W, 3.vii.2015, J. Tabell leg., GP ♂ 5480 J. Tabell); TLMF Lep 07068 (584 bp., ♂, Spain, Teruel, Albarracin. 6 km env, 1.x.2008, 40°49.5'N; 3°2.22'W, leg. & coll. L. Srnka, gen. prep. DEEUR 1000); TLMF Lep 17692 (658 bp., ♀, Spain, Tenerife, Guimar, La Ladera, 800 m, 28°18'N; 16°25'W, 23.iv.1998, leg. & coll. K. Larsen, gen. prep. DEEUR 2634); TLMF Lep 17711 (658 bp., ♀, Spain, Tenerife, Los Gigantes, 100 m, 28°17'N; 16°51'W, 8-11.i.2008, leg. & coll. K. Larsen, gen. prep. DEEUR 2807).

**Figure 69.**
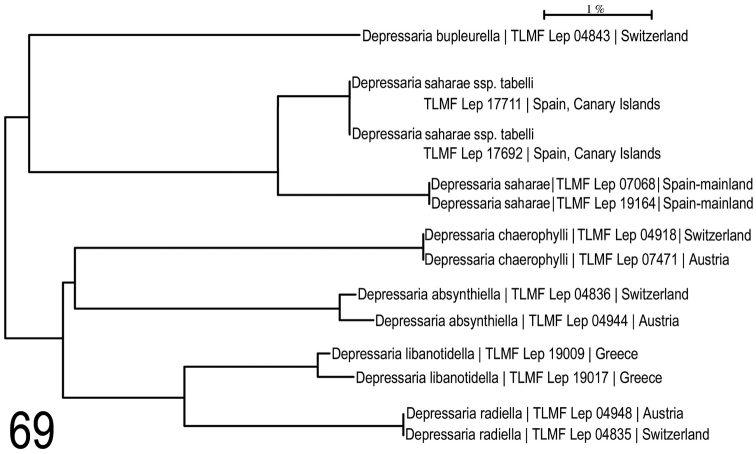
Neighbour -joining tree of *Depressaria saharae* and selected species. Associated BOLD BINs: *D. bupleurella*: BOLD:ABA1485; D. saharae ssp. tabelli ssp.n: BOLD:ADC8281; D. saharae ssp. sahaeae: BOLD:ACF8051; *D. chaerophylli*: BOLD:AAF8167; *D. absynthiella*: BOLD:ABA0596; *D. libanotidella*: BOLD:ACZ2964; *D. radiella*: BOLD:AAB6253.

**Neighbour-joining analysis** (Fig. 69) shows that *D. saharae* is a very isolated species with no obvious nearest neighbour. *Depressaria bupleurella* (BOLD:ABA1485; TLMF Lep 04843) shares a node in our NJ tree (Fig. 29), but at ~6.08-6.8% p-distance. Intraspecific variability, based on present knowledge, 0% within D. saharae ssp. saharae, 0% within D. saharae ssp. tabelli ssp.n and 2.01% between the two ssp.

**Figure 70.**
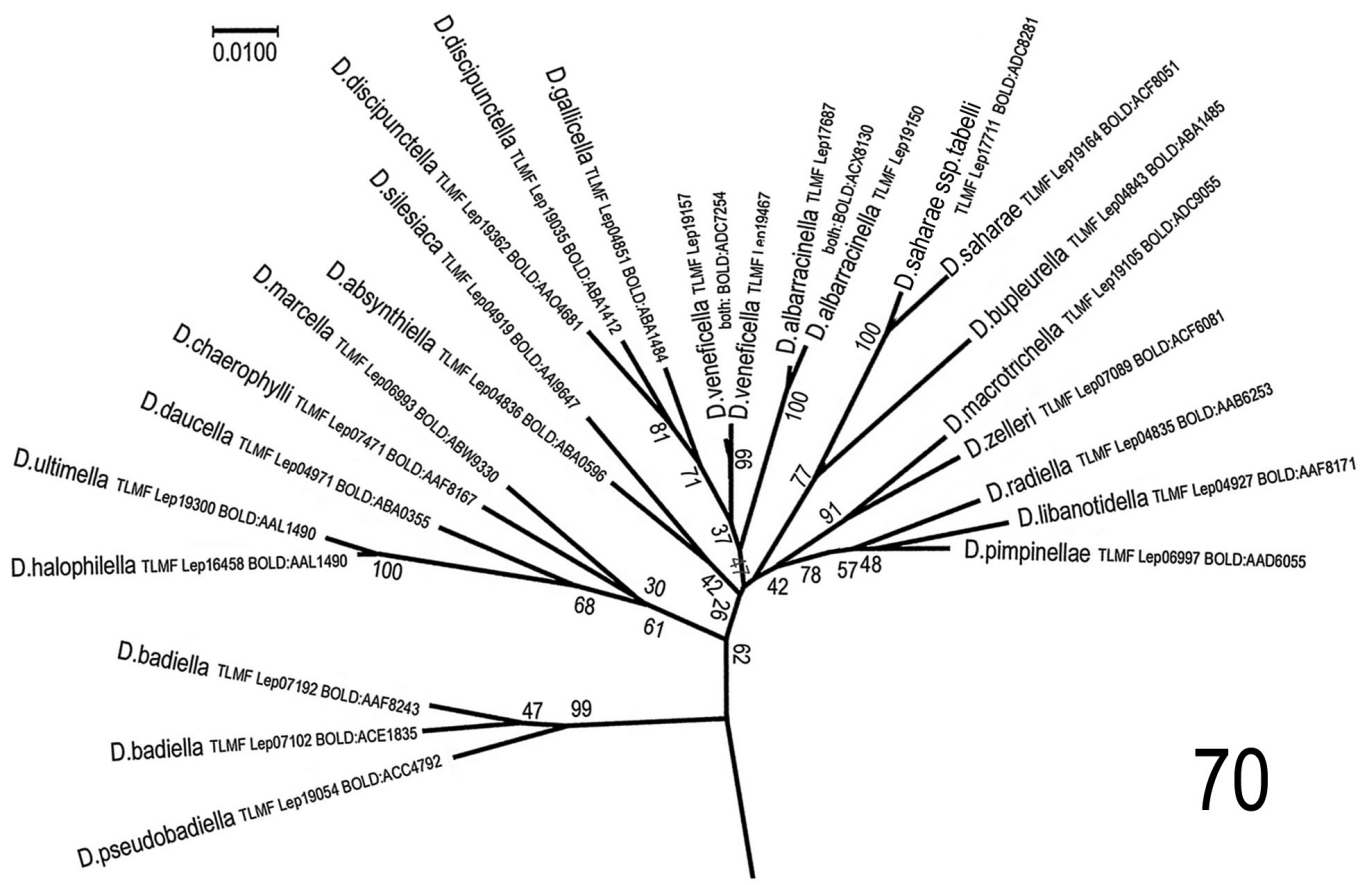
Maximum Likelihood analysis of species from the genus *Depressaria*: *D. albarracinella*, sp. n., *D. saharae* and selected species, predominately from the *D. veneficella* and *D. pastinacella* groups: In addition to the selection used for the neighbour joining tree, the following species were included: *D. absynthiella* Herrich-Schäffer, 1865 (TLMF Lep 04836, BOLD:ABA0596) *D. badiella* (Hübner,[1796]) (TLMF Lep 07102, BOLD:ACE1835 and TLMF Lep 07192, BOLD:AAF8243; *D. halophilella* Chrétien, 1908 (TLMF Lep 16458, BOLD:AAL1490); *D. macrotrichella* Rebel, 1917 (TLMF Lep 19105, BOLD:ADC9055); *D. marcella* Rebel, 1901 (TLMF Lep 06993, BOLD:ABW9330); *D. pimpinellae* Zeller, 1839 (TLMF Lep 06997, BOLD:AAD6055); *D. pseudobadiella* Nel, 2011 (TLMF Lep 19054, BOLD:ACC4792); *D. silesiaca* Heinemann, 1870 (TLMF Lep 04919, BOLD:AAI9647). The evolutionary history was inferred by using the Maximum Likelihood method based on the Tamura-Nei model. The tree with the highest log likelihood (-4818.52) is shown. Initial trees for the heuristic search were obtained automatically by applying Neighbor-Join and BioNJ algorithms to a matrix of pairwise distances estimated using the Maximum Composite Likelihood (MCL) approach, and then selecting the topology with superior log likelihood value. The tree is drawn to scale, with branch lengths measured in the number of substitutions per site. The proportion of sites where at least 1 unambiguous base is present in at least 1 sequence for each descendent clade is shown next to each internal node in the tree. The analysis involved 54 nucleotide sequences from selected species of *Agonopterix* and *Depressaria*. Only the *Depressaria*-part of the tree is shown here. Codon positions included were 1st+2nd+3rd+Noncoding. All positions containing gaps and missing data were eliminated. There were a total of 657 positions in the final dataset. Evolutionary analyses were conducted in MEGA7: Molecular Evolutionary Genetics Analysis version 7.0 for bigger datasets (Kumar, Stecher and Tamura 2015). The result is shown in radiation graphic, because in this view the evolutionary aspect is visualized better than in traditional tree.

**Maximum Likelihood analysis** (Fig. 70) shows in general the same situation. Following the conclusion of *D. bupleurella* as evolutionary neighbour based on genitalia patterns (see remarks below under Related species), this is not a surprise.

#### Related species.

Based on male genitalia, *D. saharae* belongs to the *pastinacella* group (Hannemann, 1953), named after *D. pastinacella* (Duponchel, 1838), now valid as *D. radiella* (Goeze, 1783), which is characterised by the presence of a basal process of sacculus (clavus) and the absence or near absence of a distal process of sacculus (cuiller). Within this group, genitalia of both sexes clearly show *D. bupleurella* as closest species. Neighbour Joining tree and Maximum Likelihood analysis correspond with this estimation. The close relatedness of *D. saharae* and *D. bupleurella* is also supported by biology with both species (so far only known from ssp. tabelli) feeding on *Bupleurum*.

#### Etymology.

The subspecies name, a noun in the genitive case, honours Jukka Tabell, the Finnish lepidopterologist, who collected *D. saharae* - at this time still an undescribed species - in 2015 from the Spanish mainland, and sent specimens to Peter Buchner for study. They were essential to understanding this species and led to a search for females, which were found in collections from the Canary Islands and which are here treated as a separate subspecies.

#### Distribution.

So far known only from Spain: Canary Islands (Tenerife).

#### Bionomics.

Walsingham reared one moth from larvae collected on *Bupleurum aciphyllum* (Bupleurum salicifolium ssp. aciphyllum (Webb & Berthel.) Sunding & G. Kunkel) from Canary Islands, Tenerife, Guimar. This plant is an endemic species of Macaronesia. The food-plant of D. saharae ssp. saharae is unknown, but is likely to be another species of *Bupleurum*.

#### Remarks.

The first encounter with male genitalia of *D. saharae* was a simple drawing in literature: Klimesch (1985) reports on a letter from Klaus Sattler regarding Walsingham’s bred male from Tenerife which Walsingham had referred to *D. apiella*: “….According to Dr. Sattler, NHMUK London, this specimen belongs to a species near *D. bupleurella* or to a form of *D. bupleurella*. Dissection showed differences in costa of valva and in cuiller. It must be left to a later revision of this group to decide on the final status” [translated from German]. Some males from Teruel, dissected by P. Buchner, showed this distinctive genitalia feature also. DNA barcoding supported the view that it was not a form of *D. bupleurella*, but a distinct species. As at this stage females were unknown, it remained undescribed.

In the large collection of Knud Larson, two females from Tenerife were found, which were both in external appearance and in genitalia patterns close to *D. bupleurella*, but showed a 6.36% p-distance in DNA-barcode, while barcodes show a 2% difference compared to *D. saharae* from Teruel, separating into two reciprocally monophyletic clusters. This suggested they were at least closely related, but left open the question of conspecificity. A male reared by Walsingham from Tenerife in 1907 was the key to this so far unanswered question: it has genitalia like *D. saharae* from Teruel, but in external appearance is like the females from Tenerife. The lack of genitalic separation suggests that the Teruel and Tenerife specimens are conspecific, in spite of their different external appearance. The different external appearance of the Canary Island population, in combination with the corresponding external features of both sexes of the Canary Island population justify the treatment as two separate subspecies.

## Supplementary Material

XML Treatment for Depressaria albarracinella

XML Treatment for Agonopterix carduncelli

XML Treatment for Agonopterix pseudoferulae

XML Treatment for Depressaria saharae Gaston & Vives ssp. tabelli

## Appendix

**Table:** Table with details to Barcode Index Numbers (BIN), Sample ID, Process ID and GenBank Accession for 43 BIN-conform COI-5P sequences, used for the trees in this paper.

Species	Sample ID	Process ID	BIN	GenBank Accession
*Agonopterix atomella*	BC TLMF Lep 19310	DEEUR785-16	BOLD:ABZ0059	KY754269
*Agonopterix atomella*	TLMF Lep 11536	LEATE124-13	BOLD:ABZ0059	KY754251
*Agonopterix bipunctosa*	TLMF Lep 19002	DEEUR572-16	BOLD:ABA0011	KY754228
*Agonopterix bipunctosa*	TLMF Lep 19066	DEEUR636-16	BOLD:ABA0011	KY754239
*Agonopterix bipunctosa*	TLMF Lep 19091	DEEUR661-16	BOLD:ABA0011	KY754268
*Agonopterix carduncelli* sp. n.	TLMF Lep 06978	DEEUR299-12	BOLD:ABZ7583	KY754235
*Agonopterix carduncelli* sp. n.	TLMF Lep 06994	DEEUR315-12	BOLD:ABZ7583	KY754278
*Agonopterix carduncelli* sp. n.	TLMF Lep 07015	DEEUR336-12	BOLD:ABZ7583	KY754255
*Agonopterix carduncelli* sp. n.	TLMF Lep 07017	DEEUR338-12	BOLD:ABZ7583	KY754265
*Agonopterix cluniana*	TLMF Lep 07164	PHLAI960-14	BOLD:AAM731	KY754275
*Agonopterix ferulae*	TLMF Lep 19165	DEEUR735-16	BOLD:ABW9370	KY754264
*Agonopterix ferulae*	TLMF Lep 19225	DEEUR897-16	BOLD:ABW9370	KY754248
*Agonopterix kaekeritziana*	TLMF Lep 17073	ABOLB068-15	BOLD:AAF7198	KY754233
*Agonopterix kaekeritziana*	BC TLMF Lep 19286	DEEUR761-16	BOLD:AAF7198	KY754262
*Agonopterix kaekeritziana*	TLMF Lep 14534	LASTS082-14	BOLD:AAF7198	KY754242
*Agonopterix multiplicella*	TLMF Lep 19102	DEEUR672-16	BOLD:AAF7196	KY754241
*Agonopterix oinochroa*	TLMF Lep 07109	DEEUR430-13	BOLD:ABU5789	KY754250
*Agonopterix pallorella*	TLMF Lep 07014	DEEUR335-12	BOLD:ABU5790	KY754263
*Agonopterix pallorella*	TLMF Lep 19148	DEEUR718-16	BOLD:ABA0382	KY754236
*Agonopterix pallorella*	TLMF Lep 17463	LEATI078-15	BOLD:ABU5790	KY754259
*Agonopterix pseudoferulae* sp. n.	TLMF Lep 19067	DEEUR637-16	BOLD:ACW1863	KY754266
*Agonopterix pseudoferulae* sp. n.	BC TLMF Lep 19306	DEEUR781-16	BOLD:ACW1863	KY754244
*Agonopterix pseudoferulae* sp. n.	MM24152	LEFIJ2609-15	BOLD:ACW1863	KY754238
*Agonopterix putridella*	TLMF Lep 06229	DEEUR204-11	BOLD:AAF7185	KY754256
*Agonopterix scopariella*	TLMF Lep 19263	DEEUR864-16	BOLD:ABZ0060	KY754229
*Agonopterix squamosa*	TLMF Lep 19030	DEEUR600-16	BOLD:ACF7120	KY754254
*Agonopterix squamosa*	TLMF Lep 19146	DEEUR716-16	BOLD:ACF7120	KY754249
*Agonopterix squamosa*	BC TLMF Lep 19313	DEEUR788-16	BOLD:ACF7120	KY754246
*Agonopterix squamosa*	TLMF Lep 19253	DEEUR921-16	BOLD:ACF7120	KY754240
*Agonopterix straminella*	TLMF Lep 17699	DEEUR504-15	BOLD:ACX7863	KY754276
*Agonopterix straminella*	TLMF Lep 17705	DEEUR510-15	BOLD:ABZ7581	KY754230
*Agonopterix straminella*	TLMF Lep 17721	DEEUR526-15	BOLD:ACX7863	KY754257
*Agonopterix straminella*	TLMF Lep 17727	DEEUR532-15	BOLD:ACX7863	KY754243
*Agonopterix straminella*	TLMF Lep 17731	DEEUR536-15	BOLD:ACX7863	KY754237
*Depressaria albarracinella* sp. n.	TLMF Lep 17687	DEEUR492-15	BOLD:ACX8130	KY754260
*Depressaria albarracinella* sp. n.	TLMF Lep 19062	DEEUR632-16	BOLD:ACX8130	KY754274
*Depressaria albarracinella* sp. n.	TLMF Lep 19150	DEEUR720-16	BOLD:ACX8130	KY754261
*Depressaria discipunctella*	TLMF Lep 17728	DEEUR533-15	BOLD:ABA1412	KY754252
*Depressaria discipunctella*	TLMF Lep 19035	DEEUR605-16	BOLD:ABA1412	KY754267
*Depressaria discipunctella*	BC TLMF Lep 19311	DEEUR786-16	BOLD:AAO4681	KY754270
*Depressaria discipunctella*	BC TLMF Lep 19362	DEEUR837-16	BOLD:AAO4681	KY754247
*Depressaria discipunctella*	TLMF Lep 19422	DEEUR992-16	BOLD:AAO4681	KY754234
*Depressaria eryngiella*	TLMF Lep 07107	DEEUR428-13	BOLD:ACF7124	KY754271
*Depressaria eryngiella*	BC TLMF Lep 19304	DEEUR779-16	BOLD:ACF7124	KY754277
*Depressaria libanotidella*	TLMF Lep 19009	DEEUR579-16	BOLD:ACZ2964	KY754273
*Depressaria libanotidella*	TLMF Lep 19017	DEEUR587-16	BOLD:ACZ2964	KY754253
*DepressariaDepressaria saharae*	TLMF Lep 07068	DEEUR389-13	BOLD:ACF8051	KY754279
Depressaria saharae ssp. tabelli ssp. n.	TLMF Lep 17692	DEEUR497-15	BOLD:ADC8281	KY754258
Depressaria saharae ssp. tabelli ssp. n.	TLMF Lep 17711	DEEUR516-15	BOLD:ADC8281	KY754245
*Depressaria saharae*	TLMF Lep 19164	DEEUR734-16	BOLD:ACF8051	KY754232
*Depressaria veneficella*	TLMF Lep 19157	DEEUR727-16	BOLD:ADC7254	KY754231
*Depressaria veneficella*	TLMF Lep 19467	DEEUR1037-16	BOLD:ADC7254	KY754272
